# Investigating heavy metals and other elements in *Procambarus clarkii* and environmental matrices from three wetlands of Sicily (Italy)

**DOI:** 10.1007/s11356-025-35954-y

**Published:** 2025-01-31

**Authors:** Dario Savoca, Federico Marrone, Francesco Paolo Faraone, Vittoria Giudice, Salvatore Messina, Gaetano D’Oca, Vincenzo Arizza, Antonella Maccotta, Luca Vecchioni

**Affiliations:** 1 Department of Biological, Chemical and Pharmaceutical Sciences and Technologies (STEBICEF), University of Palermo, 90123 Palermo, Italy; 2 NBFC, National Biodiversity Future Center, 90133 Palermo, Italy; 3 ARPA Sicilia, Agenzia Regionale Protezione Ambiente, UOC L2, Via Nairobi, 90129 Palermo, Italy

**Keywords:** Pollution, Trace metals, Red swamp crayfish, Bioaccumulation, Biomonitoring

## Abstract

A qualitative and quantitative analysis of 18 elements was conducted on *Procambarus clarkii* and its environmental samples to evaluate its potential as a bioindicator species. Analysis of biological samples was carried out to both assess the safety of the use of raw materials and, together with environmental samples, to assess the contamination status of the sampled sites. Significant differences highlighted by the PERMANOVA, HCA and PCA analyses confirmed the valid use of *P. clarkii* as a bioindicator of the health status of the studied ecosystem. The bioaccumulation factor (BAF) and the biotic sediment accumulation factor (BSAF), except in a few cases, reported values below the bioaccumulative criterion and showed the highest BAF values for manganese, iron and barium in the exoskeleton while the highest BSAF values concerned mercury, copper and zinc in the muscle and barium in the exoskeleton. These findings indicate that, for most of the trace elements (TEs), the extent of pollution at these sites is such that it does not result in significant bioaccumulation in the muscle and exoskeleton of *P. clarkii*. The TE concentration levels signalled mild contamination of the sampling sites, proving a good health status of the studied aquatic ecosystem. Finally, the results obtained in *P. clarkii* muscle were below the threshold limits of EU Regulation 2023/915 suggesting that these edible parts are safe for human consumption.

**Supplementary Information:**

The online version contains supplementary material available at 10.1007/s11356-025-35954-y.

## Introduction

Trace elements including heavy metals pose a serious threat to the stability of ecosystems due to their frequency and abundance, and their toxic effects. The aquatic environment generally represents a reservoir of these pollutants that can originate from industrial effluents, agricultural and urban activities, and atmospheric deposition (Goretti et al. 2016). Heavy metals have a ubiquitous distribution and are subject to bioaccumulation and biomagnification phenomena along the food chain with negative implications for wildlife and human health (Yacoubi et al. 2023).

Heavy metals can bond with organic groups by forming lipophilic molecules that can cross cell membranes and produce even more toxic compounds (e.g. tetraalkyl lead, methylmercury, methyl arsenic) or bind to non-metallic constituents of cellular molecules, such as sulphur groups in proteins, causing toxic effects (Walker et al. 2012; Briffa et al. 2020). In this context, the cycling, transformation, deposition and availability of the various forms of metals and semimetals in the aquatic ecosystem are contingent upon not only their intrinsic physicochemical natures, but also the metabolic interactions of microbial species (Frank et al. 2020).

As a result, the environmental impact of these pollutants is linked to the marked tendency of metals to accumulate in animal and plant tissues through their possible routes of exposure to contaminated food or environmental media such as water, sediment and air (Ali et al. 2019). In a given organism, its diet, stage of development, physiology and biochemical functions affect the degree of toxicity of metals and semimetals, along with their concentration and duration of exposure. In the aquatic environment, water local characteristics influence this toxicity through interactions of pH, hardness and salinity. Moreover, although the concentration of metals in the water column is generally low due to their low solubility, their tendency to precipitate to the bottom of the water bodies exposes all benthic organisms to high levels of contamination and enhance the risk of causing toxic effects (Briffa et al. 2020).

Along with the increasing concern for the effects of environmental contamination, there is a growing need to monitor and assess pollution in order to soundly plan environmental management and restoration measures (Savoca et al. 2022a).

In this context, biomonitor species are useful bioindicator tools that contain quantitative information about pollutants on the health of an ecosystem (Markert al. 1999).

The Louisiana red swamp crayfish *Procambarus clarkii* (Girard, 1852) is known as a reliable metal biomonitor species in water systems, as shown by several research (Alcorlo et al. 2006; Suárez-Serrano et al. 2010; Bellante et al. 2015; Goretti et al. 2016; Ariano et al. 2021; Selvaggi et al. 2023). The resilient physiology of *P. clarkii* enables it to tolerate low oxygen levels, high temperatures and high levels of water pollution, thereby conferring upon it a significant role in the food chain (Mistri et al. 2020). *P. clarkii* is a cambarid decapod originating from the lentic or weakly lotic water bodies of northeastern Mexico and southeastern USA. Thanks to its vivid color, pronounced euryecy and adaptability, fast growth and prolificity, the species has been largely used worldwide in aquaculture and in the aquarium trade. From the beginning of the twentieth century onwards, non-native populations of the species have been found throughout the world; these originated from escaped or intentionally released individuals (Loureiro et al. 2015). *Procambarus clarkii* is nowadays one of the most widely introduced freshwater species in the world, occurring in all continents except Oceania and Antarctica (Souty-Grosset et al. 2016). Its first occurrence in Italy dates to the late 1980s, but the species is nowadays widespread throughout the country, raising concern for the high impact it exerts on the native aquatic biota (Lo Parrino et al. 2020). As reported by Souty-Grosset et al. (2016), this decapod has long been consumed by humans, but there are numerous potential health risks to the consumer due to its ability to accumulate heavy metals and algal toxins and to transmit infectious diseases such as tularaemia.

In light of its high invasiveness and impacts on native biota, the monitoring and, when possible, eradication of the species from natural water bodies should be pursued. In this context, the possible exploitation of the *Procambarus clarkii* individuals removed in the frame of the management actions can contribute to the feasibility and economic sustainability of its local eradication. It is known that aquatic decapods have important nutritional properties, such as a high content of proteins, amino acids, carbohydrates, lipids and antioxidant activity given by omega 3 fatty acids and vitamins (Omotoso 2005; Chandrapavan et al. 2009), which make crustaceans an important food source for humans. In addition, freshwater decapods are used in pharmaceutical, biotechnological and biomedical potential applications (e.g. Andreansyah and Ridwanto 2022; Nuc et al. 2023). In fact, the non-edible portion of the crayfish, consisting of the exoskeleton, which represents a significant percentage of the total weight, usually difficult to dispose of, actually represents an important source of bioactive molecules (Mauro et al. 2022).

Today, in a circular economy, the challenge is to transform this biomass into a resource, finding protocols that allow an economic, rapid and eco-sustainable way to extract bioactive molecules of high interest from this waste material. Among these molecules, chitosan obtained from chitin is a biopolymer used in biomedical, pharmaceutical, food, agricultural and wastewater treatment (Philibert et al. 2017) and astaxanthin, a carotenoid with excellent antioxidant properties used to produce many supplements and cosmetics (Higuera-Ciapara et al. 2006; Ikeuchi et al. 2006).

For each of the possible applications mentioned above, it is essential to assess the degree of contamination of the used biological matrices that can direct to one application rather than another.

The purposes of this work were to (i) use *Procambarus clarkii* as biomonitor species by analysing 18 trace elements (including heavy metals) in biological (muscle and exoskeleton of *P. clarkii*) and environmental (water and sediment) samples in three representative areas of Sicily (Italy); (ii) compare the obtained results with those of other similar studies performed in different areas; and (iii) evaluate the elemental concentrations found for food safety.

## Material and methods

### Sampling campaign

Based on the known distribution of the species in Sicily (Faraone et al. 2017; Vecchioni et al. 2022a), three representative sites were selected for the collection of biological (individuals of *Procambarus clarkii*) and environmental (water and sediment) samples.

**Fig. 1:**
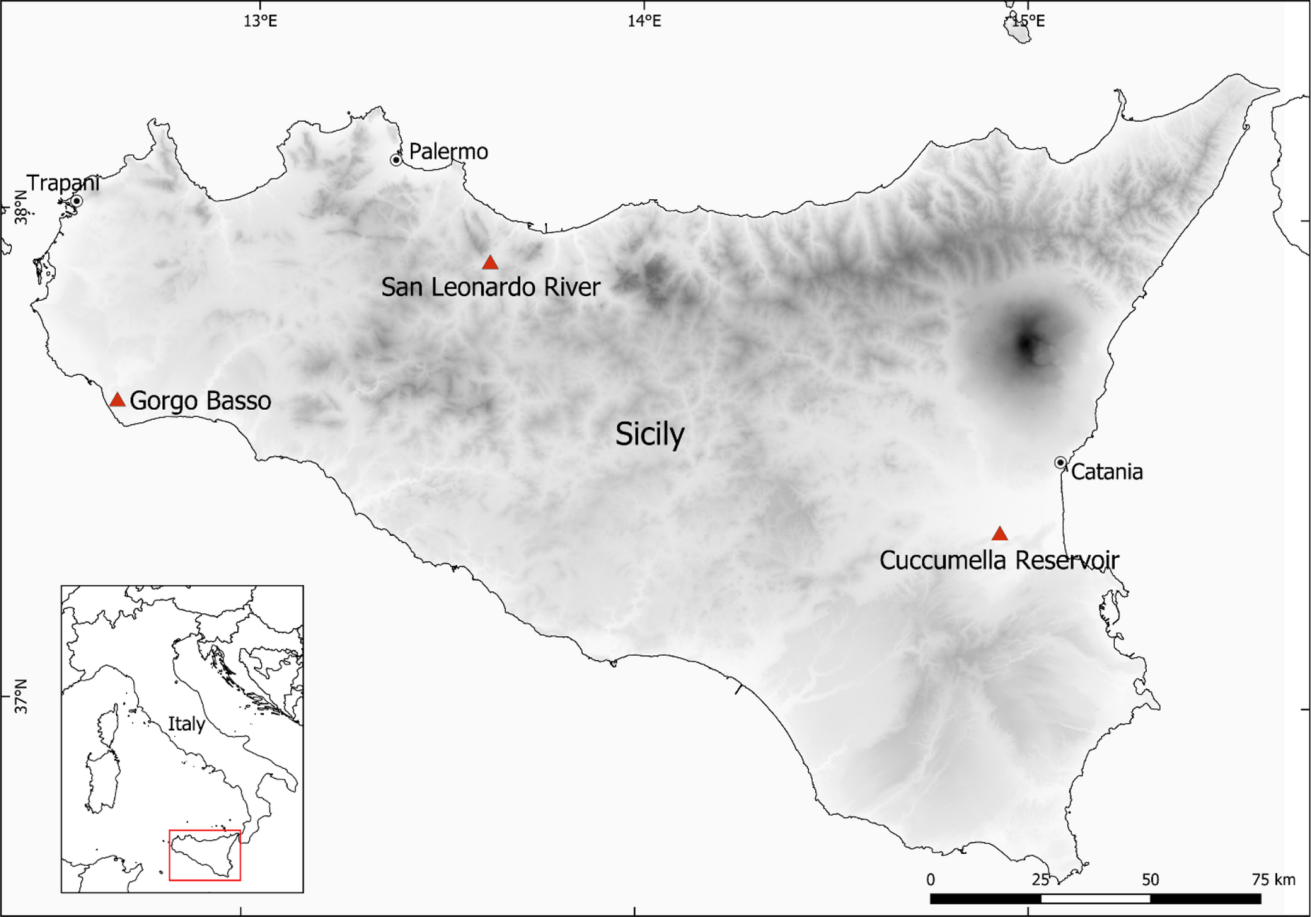
Map of sampling sites

The three selected Sicilian wetlands are Gorgo Basso (Mazara del Vallo, Trapani), San Leonardo River (Caccamo, Palermo) and Cuccumella Reservoir (Lentini, Siracusa). Further details regarding the selected sampling sites are provided in the supplementary material. The map of the sampling sites was realised using the QGIS freeware software v. 3.30.2 (QGIS Development Team, 2023; accessed on 15 January 2024) (Fig. 1).

For sampling, the summer period was chosen because it is characterised by a hydrological regime with scarce or no rainfall, higher temperatures and therefore evaporations that lead to increases in TE concentration levels. For these reasons, in our study, all the samples were taken in July 2023, and some physical and chemical parameters of the water (water temperature, pH, electric conductivity) were also contextually recorded (see Table S1 in Supporting Information). Although the three studied sites are characterised by a permanent hydroperiod, their hydrological regime heavily depends on precipitation; at the sampling dates and in the previous months, there was no rainfall, and the water bodies were close to their hydrological minimum.

In total, 500 individuals of *Procambarus clarkii* from Cuccumella Resevoir, 159 from Gorgo Basso and 266 from San Leonardo River were caught using baited hoop traps, as described in Vecchioni et al. (2020). Once collected, the specimens of *P. clarkii* were immediately transported to the laboratory in refrigerated polypropylene containers and then stored at − 20 °C. Of these 925 individuals, only the needed amount to form standardised sample pools for each site was randomly selected. In detail, 365 specimens were dissected: 183 from the Cuccumella Resevoir, 64 individuals from the San Leonardo River, 118 from the Gorgo Basso.

At the same date, from each site, three sampling points located at 50 m from each other were chosen along a transect; water samples (500 mL each) and sediment samples (500 g each) were collected in each sampling point, and then transported in the laboratory in refrigerated polypropylene containers previously washed with water collected in situ and stored at 4 °C.

Biometric data (Table S2 in Supporting Information) of all individuals of *Procambarus clarkii* were recorded with a digital calliper before performing the dissections aimed at sampling organic matrices (muscle and exoskeleton) for the preparation of the samples to be analysed.

### Experimental design and sample preparation

For each site, *Procambarus clarkii* samples were divided into five subsets (pools) (see Table S2 in Supporting Information) in order to collect high amounts of muscle and exoskeleton, and to increase the homogeneity and representativeness of measurements.

Both during sampling and sample preparation, the utmost care was spent to avoid cross-contamination or contact contamination, using new or thoroughly washed ceramics and plastics after each operation, and implementing solvent cycles such as acetone and bi-distilled water (Milli-Q water produced by a Millipore system with a resistivity of 18.2 MΩ cm).

After dissection, five muscle pool samples (edible part, without intestine) and five exoskeleton pool samples were weighed for each of the three sites for a total of 30 pool samples.

These samples were weighed, homogenised and stored in sterile polypropylene tubes and frozen at − 20 °C until freeze-drying.

The percentage of water present in the muscles (about 80%) and in the exoskeleton (about 35%) was determined according to the initial wet weight (w.w.) of the sample and the dry weight (dry weight: d.w.) after freeze-drying, according to following equation:

1 $$\% {H}_{2}O\;in\;biological\;sample =100-\frac{d.w. }{w.w. }\times 100$$

### Instrumentation and reagents

The freeze dryer used for the treatment of crayfish samples (exoskeleton and muscle) is the model Alpha 2–4 LD plus freeze-dryer, Martin Christ, Osterode am Harz, D.

The acidic mineralisation of the samples was performed with a microwave digestion system (Ethos Up Milestone connect High-Performance Microwave Digestion System) equipped with 15 containers.

The following reagents were used for the extraction procedures: nitric acid (HNO_3_ 67–69.0%, Carlo Erba Ultrapure for trace analysis), hydrogen peroxide (H_2_O_2_ 30.0%, Sigma-Aldrich for trace analysis) and hydrochloric acid HCl (34–37% Suprapure for trace analysis).

The samples were filtered with syringes equipped with cellulose acetate filters (pore size 0.45 µm, diameter 25 mm, AISIMÔ) and finally analysed in inductively coupled plasma mass spectrometry (Agilent 7800 ICP-MS with SPS4 autosampler).

The recovery percentages of the elements were obtained using certified material. For crayfish, the quality control samples were NIST-1566b (Oyster Tissue) and DORM-4 (Fish Protein). CIAC 12A Unichim, CIAC 13A Unichim and the laboratory standard 100 ppb (µg L^−1^) have been used for water analysis. As for the sediments, Meta 18 Unichim, CIAC 12 A and the laboratory standard at 100 ppb were used.

### Extraction in biological and environmental samples

About 0.6 g of each lyophilised sample was fully digested using a mixture of 8 mL HNO_3_ and 2 mL H_2_O_2_ in Teflon containers according to the EPA 3052 method.

Samples, blanks and certified reference materials (CRMs) were prepared for each mineralisation cycle.

Acid digestion was carried out using a computer-controlled heating system in Etos Up Milestone High Performance Microwawe Digestion System: from 20 to 180 °C in 15 min, maintained at 180 °C for 55 min and cooled in 10 min.

Then the samples were filtered, brought to a volume of 20 mL with bi-distilled water and analysed through ICP-MS.

In the laboratory, the water samples (100 mL each) were filtered and stabilised with 1 mL of HNO_3_. The protocol used is the one provided for surface water EPA 200.8. The treated water samples were analysed at ICP-MS.

The sediments, after arriving in the laboratory, underwent a drying pretreatment in which they were spread on filter paper to remove the water present. After 48 h, the residual moisture was analysed in duplicate as required by DM13/09/1999 met II 2.

Subsequently, about 0.6 g of each sediment sample, spiked with 6 mL HNO_3_ and 3 mL HCl, was subjected to acidic digestion with the microwave according to the methods EPA 3052 and EPA 3051 a. Finally, the samples were filtered and brought to a volume of 20 mL with bi-distilled water and analysed through ICP-MS.

### Analysis of trace elements

The analytical procedure used was in accordance with the EPA 200.8 method supplemented by EPA 6020b and EPA 3052 and three replicates were performed for each measurement. To each analytical sample, including calibration samples, a constant volume of mixed internal standard consisting of Bi, Ho, ln, 6Li, Sc, Tb, Y and Rh in HNO_3_ was added at a concentration of 200 ppb during analysis, through the peristaltic pump of the ICP-MS. In order to verify the presence of contamination in the measuring system, a series of washing solutions (rinse blank solution) have been included in the worklist for the purpose of ensuring the accuracy and reliability of the results. These comprise solutions of nitric acid (1–5% HNO₃) and hydrochloric acid (0.3–1% HCl). In the case of mercury, in order to circumvent the memory effect that may be caused by its presence in the samples, solutions of 200 ppb of gold (Au) (7% HNO₃) have been incorporated into the worklist.

The multielement calibration for the determination of the elements, including heavy metals, was carried out starting from the standard solutions of the individual analytes (initial concentration 1000 mg L^−1^ (ppm)). The analytes determined were selenium (Se), cobalt (Co), tin (Sn), vanadium (V), iron (Fe), copper (Cu), manganese (Mn), zinc (Zn), chromium (Cr), mercury (Hg), cadmium (Cd), boron (B), barium (Ba), lead (Pb), antimony (Sb), nickel (Ni), silver (Ag) and arsenic (As).

The calibration range used was 0–160 ppb for all elements except mercury, for which the range was 0–4 ppb.

Correlation coefficients showed high values (*R*^2^ = 0.9999 for each element).

A parallel analysis of procedural blanks and certified reference material (CRM) ensured quality checks and accuracy. Such analyses showed less than 10% relative standard deviation. The percentages of recovery (R) for each element in biological matrices were obtained by analysis of CRM (Table S3 in Supporting Information) according to following equation:

2 $$R =\frac{100 \times {D}_{CRM} \times \left(\frac{{V}_{CRM}}{{W}_{CRM}}\right) }{{N}_{CRM}}$$

*D*_CRM_ = measured concentration of the certified material sample (µg L^−1^).

*V*_CRM_ = final volume used for sample mineralisation—certified material (L).

*W*_CRM_ = weight of the certified material sample (g).

*N*_CRM_ = nominal concentration of the analyte in the certified material (µg g^−1^).

Accordingly, the correction factors (CF = 100/R) were calculated to quantify the concentration of each analyte in the samples. The limit of detection (LOD) and limit of quantification (LOQ) were calculated according to Savoca et al. (2022b) (Eqs. 3 and 4 respectively).

3 $$LOD={X}_{BLANK}+(3 \times {SD}_{BLANK})$$

4 $$LOQ={X}_{BLANK}+(10 \times {SD}_{BLANK})$$

X_BLANK_ = mean value of the selected analytical mass signals of ten blank measurements.

SD_BLANK_ = standard deviation of blank measurements.

The results of LOD, LOQ and recoveries for each matrix are given in Table S3 in the Supporting Information.

In order to facilitate a valid comparison of the elemental concentration levels observed in our study with those reported in other research, the concentrations expressed in wet weight were converted into dry weight, assuming an 80% water percentage in muscle samples and a 35% water percentage in exoskeleton samples. The latter values ‘marked estimated d.w.’ were calculated through the following equation:

5 $$\left[E\right] d.w.=\frac{\left[E\right] w.w.}{\left(1-\frac{\% {H}_{2}O}{100}\right)}$$

[*E*]= concentration of element

### Statistical analysis

Principal component analysis (PCA) was performed to observe similarities or differences in data (between sites and type of sample/matrix analysed) and to summarise the multivariate elemental concentration data. The element concentrations were normalised before PCA was run to remove the effect of high differences in the order of magnitude of the concentrations of the various elements.

Similarly, hierarchical cluster analysis (HCA) was used to test the similarity among the different matrices and site in relation to the contents of trace elements summarising the multivariate nature of data.

HCA was carried out using unweighted pair-group average (UPGMA) algorithms and Euclidean similarity index.

A bi-directional permutational multivariate analysis (two-way PERMANOVA) was performed to assess the differences in contamination profile between sites, or between matrices, or considering both terms together (interaction between matrices and sites). Each term in the analysis was tested by 9999 random permutations based on Euclidean distance. The experimental design included two factors (sites (SLR, GB, CR, three levels, fixed and orthogonal) and matrices (muscle, exoskeleton, sediment, water, four levels, fixed and orthogonal)) and 18 variables corresponding to the elements analysed.

For statistical analysis, values below the limit of detection were substituted with the corresponding LOD/2. All statistical analyses were performed using PAST 4.04 software (Hammer et al. 2001).

For each site and matrix, the bioaccumulation potentials have been calculated: the bioaccumulation factor (BAF) and the biota sediment accumulation factor (BSAF). These bioaccumulation descriptors have been investigated to assess the element’s bioaccumulatibility and the resulting bioaccumulation capacity of biological tissues from environmental matrices. Both factors have been determined according to the equations present in a previous works (Savoca et al. 2022b, 2023).

The BAF has been calculated as the ratio between the mean concentrations of the element present in the muscle or exoskeleton and the average concentrations of the same element recorded in water. Similarly, the BSAF was calculated in the same way as the BAF except for the use of the sediment concentrations at the denominator instead of the water concentrations.

## Results

The average concentration levels of the elements of the different analysed samples are given in Tables 1 and 2; the individual analyses by pool (biological samples) or by sample (environmental samples) are reported in Table S4 in the Supporting Information.

**Table 1:** Average levels (µg g^−1^ d.w. or µg L^−1^) ± SD measured for each of the matrices at the three sites (*SLR* San Leonardo River, *GB* Gorgo Basso, *CR* Cuccumella Reservoir)

Site	Matrix	Sb	As	B	Cd	Co	Cr	Fe	Mn	Hg	Ni
**SLR**	Muscle (*n* = 5)	<	0.92 ± 0.57	1.23 ± 0.22	<	<	0.43 ± 0.20	94.02 ± 69.09	11.52 ± 7.09	0.223 ± 0.102	2.11 ± 3.22
	Exoskeleton (*n* = 5)	<	0.41 ± 0.14	4.46 ± 0.61	<	1.60 ± 0.56	1.13 ± 0.21	671.10 ± 291.76	528.63 ± 183.04	0.015 ± 0.005	1.19 ± 0.35
	Sediments (*n* = 3)	<	6.51 ± 0.90	11.53 ± 7.48	0.119 ± 0.025	7.30 ± 2.56	32.33 ± 22.08	21,158.99 ± 5254.55	385.67 ± 58.96	0.014 ± 0.004	15.61 ± 4.87
	Water (*n* = 3)	<	<	304.82 ± 19.64	0.005* ± 0.006	1.39 ± 0.11	<	14.94* ± 22.24	0.49* ± 0.60	<	1.30* ± 2.00
**GB**	Muscle (*n* = 5)	<	2.43 ± 0.37	1.59 ± 0.27	<	<	0.15 ± 0.13	29.08 ± 8.03	1.11 ± 0.55	0.297 ± 0.088	0.19 ± 0.20
	Exoskeleton (*n* = 5)	<	0.86 ± 0.11	6.11 ± 1.07	<	0.63 ± 0.04	0.61 ± 0.15	189.29 ± 28.73	68.63 ± 12.90	0.009 ± 0.002	0.30 ± 0.14
	Sediments (*n* = 3)	0.55 ± 0.05	16.47 ± 0.81	16.81 ± 2.75	0.132 ± 0.013	4.54 ± 0.62	17.50 ± 1.06	10,593.61 ± 515.39	192.69 ± 22.62	0.036 ± 0.003	7.25 ± 0.36
	Water (*n* = 3)	0.37 ± 0.26	10.97 ± 0.44	634.25 ± 11.75	0.004 ± 0.005	<	<	19.68 ± 3.87	1.37 ± 2.11	<	<
**CR**	Muscle (*n* = 5)	<	1.90 ± 0.14	1.42 ± 0.19	0.005 ± 0.006	<	0.54 ± 0.28	91.40 ± 64.72	16.40 ± 9.99	0.363 ± 0.082	0.73 ± 0.26
	Exoskeleton (*n* = 5)	<	0.50 ± 0.09	5.02 ± 0.22	<	0.82 ± 0.16	0.74 ± 0.17	351.47 ± 122.65	171.96 ± 57.49	0.009 ± 0.001	0.85 ± 0.08
	Sediments (*n* = 3)	<	9.69 ± 2.20	55.56 ± 6.63	0.276 ± 0.037	19.34 ± 2.35	86.51 ± 11.02	40,552.88 ± 4096.80	948.91 ± 145.89	0.030 ± 0.004	44.92 ± 2.38
	Water (*n* = 3)	<	4.04 ± 0.47	419.79 ± 57.07	0.011 ± 0.009	<	<	<	0.43* ± 0.49	<	<

*Element found only in one pool; < : below the limit of quantification; *NA* not analysed. *n* = number of pools and number of samples for environmental matrices

**Table 2:** Average levels (µg g−1 d.w. or µg L−1) ± SD measured for each of the matrices at the three sites (SLR San Leonardo River, GB Gorgo Basso, CR Cuccumella Reservoir)

Site	Matrix	**Pb**	**Cu**	**Se**	**V**	**Zn**	**Ag**	**Sn**	**Ba**
**SLR**	Muscle (*n* = 5)	Pb	Cu	Se	V	Zn	Ag	Sn	Ba
	Exoskeleton (*n* = 5)	<	52.41 ± 21.53	1.19 ± 0.44	0.08* ± 0.08	88.96 ± 8.25	<	<	0.74 ± 0.40
	Sediments (*n* = 3)	0.11 ± 0.14	29.53 ± 4.30	0.41 ± 0.03	1.12 ± 0.36	15.90 ± 4.64	<	<	146.34 ± 24.99
	Water (*n* = 3)	6.59 ± 3.37	8.34 ± 3.07	1.20 ± 0.54	40.02 ± 23.71	44.38 ± 17.14	<	0.72 ± 0.67	143.91 ± 66.06
**GB**	Muscle (*n* = 5)	<	<	<	<	<	NA	<	34.79 ± 2.58
	Exoskeleton (*n* = 5)	<	27.07 ± 5.53	0.51 ± 0.04	<	106.25 ± 22.77	<	<	5.85 ± 2.94
	Sediments (*n* = 3)	<	12.24 ± 1.40	0.27 ± 0.13	0.41 ± 0.08	14.95 ± 3.23	<	<	142.09 ± 13.89
	Water (*n* = 3)	12.63 ± 2.51	25.46 ± 1.41	0.83 ± 0.05	33.47 ± 1.59	40.51 ± 5.85	<	0.58 ± 0.07	47.31 ± 6.54
**CR**	Muscle (*n* = 5)	<	<	<	0.94 ± 0.68	<	NA	<	32.41 ± 0.50
	Exoskeleton (*n* = 5)	<	53.57 ± 12.14	1.09 ± 0.07	<	117.14 ± 8.96	<	<	5.85 ± 2.94
	Sediments (*n* = 3)	<	19.55 ± 5.60	0.38 ± 0.04	0.95 ± 0.35	12.54 ± 2.77	<	<	537.45 ± 120.68
	Water (*n* = 3)	23.94 ± 4.31	30.03 ± 1.47	2.34 ± 0.30	126.10 ± 15.73	123.32 ± 5.67	<	2.40 ± 0.35	265.01 ± 28.49
		<	<	<	6.38 ± 4.14	<	NA	<	49.78 ± 54.28

*Element found only in one pool; < : below the limit of quantification; NA not analysed. n = number of pools and number of samples for environmental matrices

Antimony, silver, tin, cadmium (except for a contaminated muscle sample) and lead (except for a contaminated exoskeleton sample) are all below the limit of quantification in biological samples.

Arsenic, mercury, nickel, copper, selenium and zinc showed higher mean values in muscle samples compared to those of exoskeleton (see Tables 1 and 2).

Boron, iron, manganese, vanadium, barium and cobalt showed higher mean values in the exoskeleton) compared to muscle (see Tables 1 and 2).

Regarding chemical analyses on environmental matrices, except for boron, for all elements and all sites, the concentration levels are at least three orders of magnitude higher in sediment samples than in water (see Tables 1 and 2).

Environmental samples taken from each site in the three different areas showed similar concentrations. Except for arsenic and mercury, the sediments from the Cuccumella Reservoir were more contaminated (see Tables 1 and 2) than those from San Leonardo River and Gorgo Basso.

Conversely, apart from cobalt, nickel, vanadium and barium, the water samples from Gorgo Basso had higher concentrations of the elements analysed than the other sites.

## Discussion

The PERMANOVA analysis showed significant differences between contamination profiles observed both between sites (PERMANOVA; *p* = 0.0001), between matrices (PERMANOVA; *p* = 0.0001) and considering both factors (interaction between sites and matrices) (PERMANOVA; *p* = 0.0001). The results substantiate the hypothesis that the distinct natures of the matrices result in differential distributions of trace elements. Additionally, the analysis reveals that the contamination levels at the three sites exhibit notable discrepancies, which are predominantly influenced by the elevated concentration levels observed in the biological matrices.

**Fig. 2:**
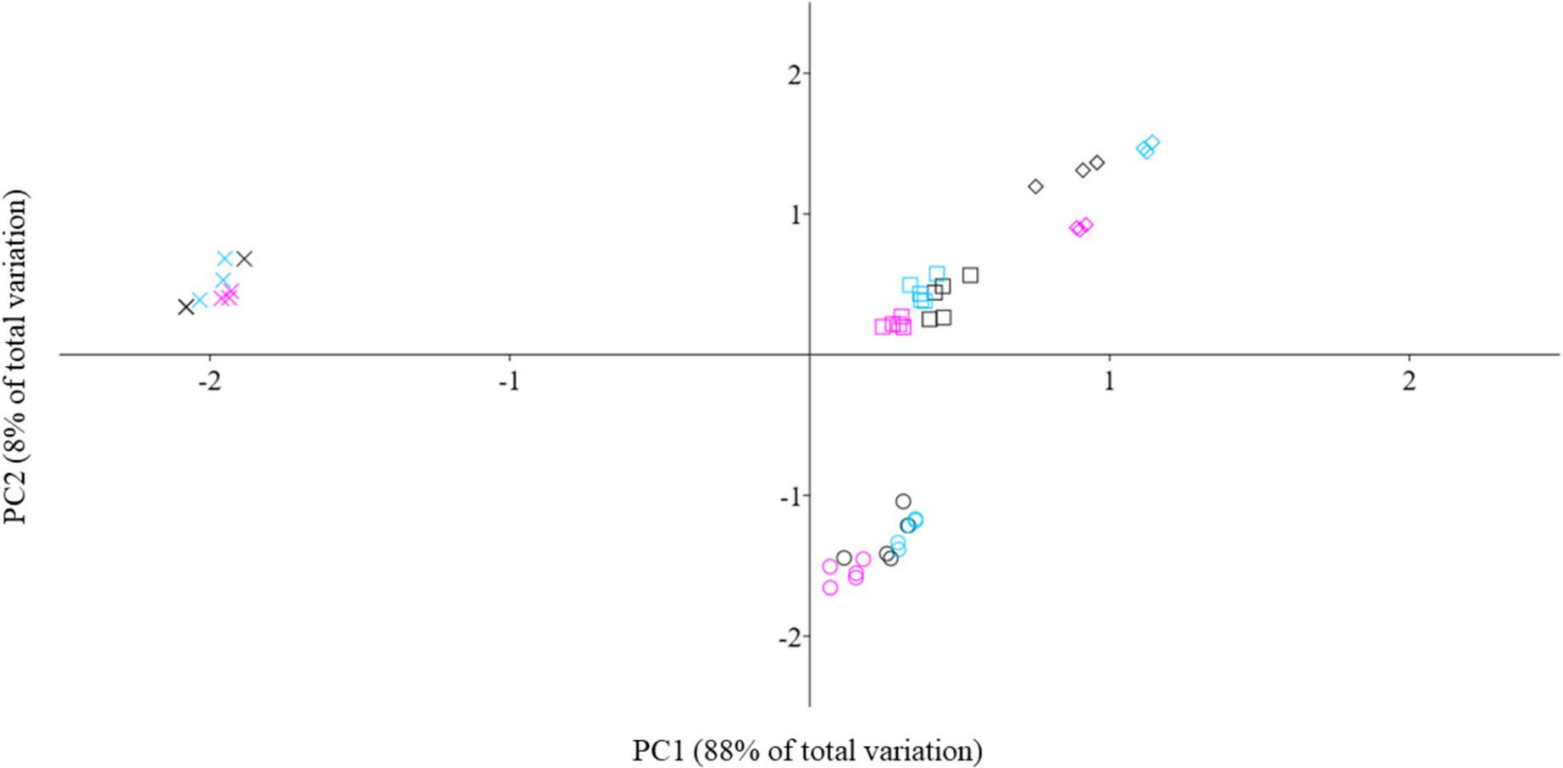
Principal component analysis (PCA). Muscle (dots), exoskeleton (squares), sediment (rhombus) and water (X) of samples from San Leonardo River (black), Gorgo Basso (fuchsia) and Cuccumella Reservoir (sky blue)

Similarly, the PCA analysis showed a clear separation in terms of TEs contamination profile between different matrices (symbols) and, to a lesser extent, between sites (colours) (Fig. 2).

**Fig. 3:**
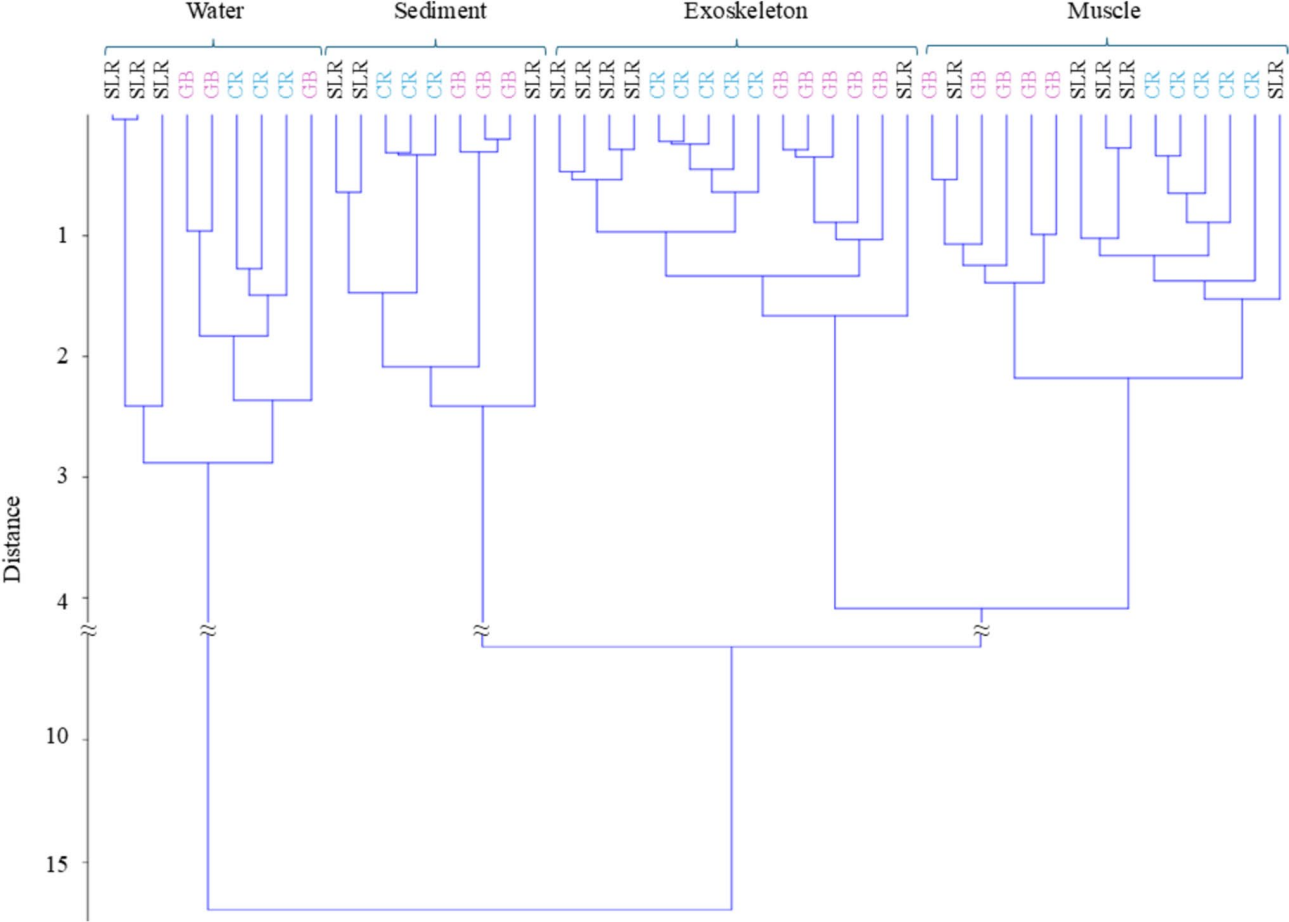
Dendrogram obtained by HCA based on the Euclidean distance between matrices (specified on the top) belonging from San Leonardo River (SLR in black), Gorgo Basso (GB in fuchsia) and Cuccumella Reservoir (CR in sky blue)

The similarity in elemental composition profile between the sediment and exoskeleton samples observed in the PCA may be attributed to the close contact between the two matrices during the life of *P. clarkii*. Furthermore, the HCA analysis demonstrates that, in the majority of cases, different clusters are formed between sites within the same matrix. Both for the exoskeleton (see number 1 in Fig. 3) and for the muscular tissues (see number 18 in Fig. 3), only one pool out of 15 is associated with sites other than its belonging.

Based on the observed differences between sites in the elemental contamination profile in muscle and exoskeleton, PCA, HCA and PERMANOVA analyses confirm that *Procambarus clarkii* is a good bioindicator of the state of environmental contamination.

It is important to note that environmental matrices act as a local source of trace elements, which can influence the bioaccumulation of these elements in organisms. In this study, the elevated levels of barium present in the water (49.78 µg L^−1^) and sediment (265.01 mg kg^−1^) at Cuccumella Reservoir may have contributed to the elevated concentrations observed in individuals of *P. clarkii*, particularly in the exoskeleton, which exhibited higher levels (537.45 mg kg^−1^) compared to the other sites (142.09 mg kg^−1^ in GB and 146.34 mg kg^−1^ in SLR). Furthermore, given the vicariance between barium and calcium, it is plausible that the exoskeleton has accumulated considerable quantities of Ba through direct contact with the sediment and water.

Similarly, the highest concentrations of Mn were observed at the SLR site, where the exoskeleton and muscle of *P. clarkii* also exhibited higher levels of this element than at other sites. In other studies, the impact of contamination in environmental matrices on levels in biota has also been verified. For example, Gedik et al. (2017) observed that the arsenic content in the gill tissue of *P. clarkii* collected at various sampling sites showed a positive correlation with the concentration of As in pond water. In a similar study, the concentrations of As and Pb in the sediment and tissue of *P. clarkii* were found to be significantly correlated with the prevalence of intensive agricultural activities, particularly the extensive use of fertilisers and pesticides (Bellante et al. 2015). However, despite the relatively low concentrations of Ni observed in sediment samples, the concentrations of Ni detected in muscle tissues were found to be within the concentration range previously observed in heavily polluted waters (Bellante et al. 2015). In fact, additional factors may be considered, including the selective bioaccumulation of certain elements due to competitive interactions between them or other environmental variables such as water and sediment mixing. In this context, Selvaggi et al. (2023) have recently observed a close relationship between fluctuations in precipitation-related hydrological levels and contamination of sediment trace elements.

In general, the discrepancies in contamination profiles observed in organisms from different geographical regions are often attributable to the specific environmental sources of contamination to which they are exposed.

Several papers have studied the trace elements in the muscle and exoskeleton tissues of *P. clarkii* of which Table 3 shows the concentration levels of different elements.

**Table 3:** Comparison of average elemental concentration levels expressed in mg kg^−1^ d.w. in muscles of *Procambarus clarkii* from present work with other regions. The levels of Sb, B and Sn have not been added to the table as they have not been investigated in the other studies reported

Location	Matrix	As	Cd	Co	Cr	Fe	Mn	Hg	Ni	Pb	Cu	Se	V	Zn	Ba	Ag
**Present study**	Muscle (*n* = 365)	1.75	0.003	<	0.37	71.50	9.68	0.294	1.01	<	44.35	0.93	0.06	104.12	2.40	<
	Exoskeleton (*n* = 365)	0.59	<	1.02	0.83	403.95	256.41	0.011	0.78	0.07	20.44	0.36	0.83	14.46	203.02	<
**South-Western Sicily (Italy)**^**1**^	Muscle (*n* = 50)	1.79	0.01	NA	0.81	NA	NA	NA	0.58	0.18	17.3	NA	0.08	74.9	NA	NA
**Tuscany (Italy)**^**2**^	Muscle (*n* = 10)	NA	2.4	NA	NA	NA	NA	NA	NA	0.9	187	NA	NA	156	NA	NA
**Umbria (Italy)**^**2**^	Muscle (*n* = 10)	NA	2.2	NA	NA	NA	NA	NA	NA	2.0	27	NA	NA	98	NA	NA
**Umbria (Italy)**^**3**^	Muscle* (*n* = 71)	NA	0.025	0.125	10	55.25	7.4	0.0002	10.25	NA	33.25	0.3	NA	66	NA	0.06
**Campania (Italy)**^**4**^	Muscle* (*n* = 60)	2.71	<	NA	0.183	NA	NA	<	NA	<	24.23	NA	NA	433.79	NA	NA
**Sharkia** **Governorate (Egypt)**^**5**^	Muscle* (*n* = 96)	36.9	2.89	NA	NA	NA	NA	8.55	NA	1.65	NA	NA	NA	NA	NA	NA
**River Nile (Egypt)**^**6**^	Muscle (*n* = 100)	NA	NA	NA	0.40	NA	4.89	NA	0.45	NA	4.03	NA	NA	NA	NA	0.31
	Exoskeleton (*n* = 100)	NA	NA	NA	1.26	NA	58.65	NA	2.97	NA	8.48	NA	NA	NA	NA	2.29
**River Nile (Egypt)**^**7**^	Muscle* (*n* = 40)	NA	5.5	NA	NA	NA	NA	NA	NA	38.1	NA	NA	NA	NA	NA	NA
	Exoskeleton* (*n* = 40)	NA	0.11	NA	NA	NA	NA	NA	NA	18	NA	NA	NA	NA	NA	NA
**River Nile (Egypt)**^**8**^	Muscle (*n* = 40)	0.69	1.34	1.01	NA	100.93	NA	NA	NA	3.21	NA	NA	NA	36.90	NA	NA
	Exoskeleton (*n* = 40)	2.73	7.82	8.56	NA	185.89	NA	NA	NA	39.21	NA	NA	NA	18.43	NA	NA
**Jiangsu Province China)**^**9**^	Muscle (*n* = 30)	1.13	0.024	NA	0.714	NA	16	0.222	NA	0.241	17.3	NA	NA	95.9	NA	NA
**Cultivated pound in Hubei province (China)**^**10**^	Muscle*(*n*≈100)	2.85	2.25	NA	65.2	1359.7	731.5	NA	NA	6.35	78.41	NA	NA	71.8	85.8	NA
	Exoskeleton* (*n*≈100)	0.17	0.72	NA	17.92	120.80	9.55	NA	NA	2.05	18.94	NA	NA	32.46	2.58	NA
**Uncultivated pound in Hubei province (China)**^**10**^	Muscle (*n*≈100)	0.7	5.615	NA	24.55	623.45	33.55	NA	NA	9.4	42.4	NA	NA	98.45	25.55	NA
	Exoskeleton (*n*≈100)	0.91	1.82	NA	9.62	931.31	201.57	NA	NA	3.74	23.45	NA	NA	27.83	31.91	NA
**Louisiana** **(USA)**^**11**^	Muscle (*n*≈60)	NA	0.06	NA	NA	NA	NA	NA	NA	3.50	29.79	NA	NA	59.1	NA	NA
	Exoskeleton (*n*≈60)	NA	0.06	NA	NA	NA	NA	NA	NA	1.34	15.76	NA	NA	18.09	NA	NA

^1^Bellante et al. 2015

^2^Goretti et al. 2016

^3^Selvaggi et al. 2023

^4^Ariano et al. 2021

^5^Khater et al. 2024

^6^Abbas et al., 2023

^7^Shaaban et al. 2017

^8^El-Aziz, 2022

^9^Anandkumar et al. 2020

^10^Xiong et al. 2020

^11^Gedik et al. 2017

*The concentration values for these samples were transformed from w.w. to d.w. *N* = number of individuals analysed. < : below the limit of quantification

Comparing the results obtained in this work with those found in other papers, it is observed that there are differences in TEs concentration profiles.

Bellante et al. (2015) analysed eight TEs in muscle samples of *Procambarus clarkii* captured at two sites in western Sicily (Gorgo Medio and Preola Lake), which are located near one of the sites investigated by us (Gorgo Basso), and hydrologically connected to it. Comparing the concentrations found by Bellante et al. (2015) with our observations, the levels are comparable. Arsenic concentrations were similar (average present study = 2.43 mg kg^−1^; average Preola Lake = 1.93 mg kg^−1^, average Gorgo Medio = 1.62 mg kg^−1^) as well as those of Cd, below the limit of detection in all samples of this study, while in Bellante et al. (2015) they were detected at low concentrations only in a sample from Preola Lake (0.01 mg kg^−1^) and five samples from the Gorgo Medio (range 0.01–0.03 mg kg^−1^). On average, the chromium found in the specimens of Gorgo Basso (0.15 mg kg^−1^) was slightly lower than that recorded by Bellante et al. (2015) (0.93 mg kg^−1^ in Preola Lake and 0.68 mg kg^−1^ in the Gorgo Medio). Similarly, the average nickel concentrations were lower (present study = 0.19 mg kg^−1^; Preola Lake = 0.73 mg kg^−1^; Gorgo Medio = 0.97 mg kg^−1^). Average levels of Cu and Zn in our work were slightly higher (Cu = 27.07 mg kg^−1^ and Zn 106.25 mg kg^−1^) than those found by Bellante et al. (2015) in the Preola Lake (average Cu = 12.61 mg kg^−1^; average Zn = 75.49 mg kg^−1^) and in the Gorgo Medio (average Cu = 23.04 mg kg^−1^; average Zn = 74.18 mg kg^−1^).

Both Pb and V in the present work were below the limit of detection while in the Preola Lake (average Pb = 0.1 mg kg^−1^; average V = 0.05 mg kg^−1^) and in the Gorgo Medio (average Pb = 0.34 mg kg^−1^; average V = 0.12 mg kg^−1^) they were higher.

The levels found in our study are lower than those found by Goretti et al. (2016) in some specimens of *Procambarus clarkii* from Lake Trasimeno and from an industrial area of central Italy. In this work, higher levels of TEs in both *Procambarus clarkii* biological samples and sediments were detected during the summer period than in other seasons (Goretti et al. 2016). In particular, they found a mean concentration of cadmium in the abdominal muscle of 2.3 mg kg^−1^ while in the three monitoring sites of our work, cadmium in *P. clarkii* muscle was below the limit of quantification except for Cuccumella Reservoir (Cd average concentrations, 0.005 mg kg^−1^).

Cadmium was also not detected in Campania (Ariano et al. 2021), while in other countries, particularly Egypt (Shaaban et al. 2017, Khater et al. 2024) and China (Mo et al. 2022), the levels of cadmium were at least two orders of magnitude higher than those observed in the Cuccumella Reservoir.

In the three Sicilian sites analysed in the frame of current study, the lead in the muscle was below the limit of quantification unlike what was observed by Goretti et al. (2016) in the muscle of *P. clarkii* of the central Italy industrial site (average = 0.9 mg kg^−1^) and in those of Lake Trasimeno (average = 2.0 mg kg^−1^).

Lead was below the limit of detection also in the individuals caught in Campania (Ariano et al. 2021), unlike the low levels recorded in Spain (Suárez-Serrano et al. 2010) and the high levels observed in some specimens caught on the banks of the Nile (Egypt), on average up to about 38 mg kg^−1^ estimate d.w. (Shaaban et al. 2017).

Zinc and copper showed respectively average concentrations of 156 and 187 mg kg^−1^ in the muscles of individuals of the central Italy industrial site and 98 and 27 mg kg^−1^ in those of Lake Trasimeno (Goretti et al. 2016). In our research, the averages obtained for zinc and copper in the three Sicilian sites (SLR 88.96 mg kg^−1^; GB 106.25 mg kg^−1^; CR 117.14 mg kg^−1^) were similar to what was observed in central Italy by Goretti et al. (2016) while, for copper (SLR 52.41 mg kg^−1^; GB 27.07 mg kg^−1^; CR 53.57 mg kg^−1^), they were of the same order of magnitude as those recorded in Lake Trasimeno and lower than those of the industrial site. Zinc and copper levels were similar to those observed in Spain (Sánchez López et al. 2004; Alcorlo et al. 2006; Suárez-Serrano et al. 2010) and Louisiana (USA) (Hogan et al. 2007).

Copper and zinc are essential micronutrients, regulated through physiological processes (Orecchio et al. 2014). However, they may be toxic at levels above the physiological range (Yacoubi et al. 2024). The levels found for these two elements are similar to those reported in the literature (Table 3) and are not particularly concerning. In our work, As (average of three sites = 1.75 mg kg^−1^), Hg (average of three sites = 0.29 mg kg^−1^) and Cr (average of three sites = 0.37 mg kg^−1^) were slightly higher than those found in Campania (As average of two sites = 0.54 mg kg^−1^; Cr: average of two sites = 0.05 mg kg^−1^; Hg: average of two sites =  < LOQ (Ariano et al. 2021).

Comparing these three elements with other studies in the literature, in our samples, arsenic levels were similar to those observed by Suárez-Serrano et al. (2010) and lower than those recorded by Mo et al. (2022) and Khater et al. (2024). Mercury values were lower than those recorded in Spain by Suárez-Serrano et al. (2010) (Hg, range 0.22–3.1 mg kg^−1^) and by Higueras et al. (2006) (Hg, range 2.4–9.1 mg kg^−1^), in Louisiana (USA) by Hogan et al. (2007) (Hg, range 60–70 mg kg^−1^) and by Mo et al. (2022) (Hg, range 0.1–0.3 mg kg^−1^ estimated d.w.) and Zhang et al. (2023) (Hg, 0.5 mg kg^−1^) in several lakes of China. In these studies, the highest levels of mercury were also found in muscle compared to other tissues. Inorganic mercury can be converted to methylmercury by methylation by sulfate-reducing bacteria in sediments (Bian et al. 2023). It has been reported that approximately 90% of the total mercury in crayfish is in the form of methylmercury, which accumulates in proteins as a consequence of its affinity for the sulphur-containing amino acid cysteine (Bian et al. 2023). Therefore, it can be posited that the elevated concentration of Hg in abdominal muscles is attributable to the relatively high protein content of these muscles and the higher level of methylmercury present in the environment.

Chromium concentrations were lower than those found in muscle samples of *Procambarus clarkii* in Spain (average of seven sites = 1.94 mg kg^−1^) (Suárez-Serrano et al. 2010) and in China (average of seven sites = 1.1 mg kg^−1^ estimated d.w.) (Mo et al. 2022).

In another study conducted on Lake Trasimeno at other sampling sites, the following elements were analysed in the *Procambarus clarkii* muscle: Cr, Mn, Fe, Co, Ni, Cu, Zn, Ag, Cd, Pb, Hg (Selvaggi et al. 2023). Moreover, the seasonal and sex variables of the animals were considered. The highest concentrations were found in the summer, while no significant differences were observed in the variations in the bioaccumulation of the elements concerning the sex of the individuals (Selvaggi et al. 2023).

The comparison was carried out considering the analysis carried out in the summer by Selvaggi et al. (2023) and found that, for different elements in our work, the concentration was lower: Cr (average of Sicilian sites, 0.37 mg kg^−1^ vs 32.5 mg kg^−1^ estimated d.w.), Fe (average of Sicilian sites, 71.5 mg kg^−1^ vs 182 mg kg^−1^ estimated d.w.), Co (average of Sicilian sites, < LOQ vs 0.2 mg kg^−1^ estimated d.w.), Ni (average of Sicilian sites, 1.01 mg kg^−1^ vs 44 mg kg^−1^ estimated d.w.); while for the rest it was similar: Ag (average of Sicilian sites, < LOQ vs 0.04 mg kg^−1^ estimated d.w.), Cd (average of Sicilian sites, < LOQ vs 0.02 mg kg^−1^ estimated d.w.), Pb (average of Sicilian sites, < LOQ vs 0.14 mg kg^−1^ estimated d.w.), Hg (average of Sicilian sites, 0.29 mg kg^−1^ vs 0.22 mg kg^−1^ estimated d.w.); or slightly higher in the case of three essential elements: Mn (average Sicilian sites, 9.68 mg kg^−1^ vs 6 mg kg^−1^ estimated d.w.), Cu (average Sicilian sites, 44.35 mg kg^−1^ vs 33.5 mg kg^−1^ estimated d.w.), Zn (average Sicilian sites, 104.12 mg kg^−1^ vs 69 mg kg^−1^ estimated d.w.).

In general, regarding non-essential trace elements, European Union legislation (EC Regulation No 1881/2006 updated to EU Regulation 2023/915) on food safety clearly lays down limit values for crustacean muscles for human consumption. In particular, the maximum levels for wet weight (w.w.) are 0.5 mg kg^−1^ for cadmium, lead and mercury, corresponding to 2.5 mg kg^−1^ in dry weight.

Therefore, in the present work, none of the samples exceeds the threshold limits from the European legislation. For these elements, muscle concentration levels are acceptable for food purposes.

However, evidence indicates that other crustacean parts, such as the hepatopancreas, also exhibit elevated levels of contamination (Ariano et al. 2021; Zhou et al. 2024). Consequently, it is essential to consider the specific anatomical region consumed, as potential contamination risks may arise. Similarly, risks may emerge if individuals of *P. clarkii* are consumed in their entirety by other predators or humans (Rios et al. 2013).

In order to investigate the TE bioaccumulative potential in the muscle and exoskeleton of *P. clarkii*, the bioaccumulation descriptors BAF and BSAF are reported in Table 4.

**Table 4:** Bioaccumulation descriptors for trace elements calculated in muscle or exoskeleton of *P. clarkii*

Factors	Matrix	Site	As	B	Cd	Co	Cr	Fe	Mn	Hg	Ni	Pb	Cu	Se	V	Zn	Ba
**BAF**	Muscle	SLR	ND	4.04	ND	ND	ND	**6293**.86	**23,327**.48	ND	**1620**.05	ND	ND	ND	ND	ND	21.33
		GB	221.80	2.51	ND	ND	ND	**1477**.87	808.56	ND	ND	ND	ND	ND	ND	ND	18.82
		CR	469.23	3.38	442.75	ND	ND	ND	**37,792**.63	ND	ND	ND	ND	ND	ND	ND	117.39
	Exoskeleton	SLR	ND	14.62	ND	1151.08	ND	**44,922**.55	**1,070,819**.72	ND	912.81	ND	ND	ND	ND	ND	**4206**.56
		GB	78.19	9.63	ND	ND	ND	**9619**.11	**50,085**.62	ND	ND	ND	ND	ND	444.44	ND	**4385**.00
		CR	122.75	11.95	ND	ND	ND	ND	**396,216**.59	ND	ND	ND	ND	ND	148.50	ND	**10,796**.18
**BSAF**	Muscle	SLR	0.14	0.11	ND	ND	0.01	< 0.005	0.03	**15.18**	0.13	ND	**6.28**	0.99	< 0.005	**2.00**	0.005
		GB	0.15	0.09	ND	ND	0.01	< 0.005	0.01	**8.25**	ND	ND	**1.06**	0.62	ND	**2.62**	0.01
		CR	0.20	0.03	ND	ND	0.01	< 0.005	0.01	**12.22**	ND	ND	**1.78**	0.46	ND	0.95	0.02
	Exoskeleton	SLR	0.06	0.39	ND	0.22	0.03	0.03	**1.37**	**1.01**	0.08	0.02	**3.54**	0.34	0.03	0.36	**1.02**
		GB	0.05	0.36	ND	0.14	0.03	0.02	0.36	0.261	0.04	ND	0.48	0.33	0.01	0.37	**3.00**
		CR	0.05	0.09	ND	0.04	0.01	0.01	0.18	0.297	0.02	ND	0.65	0.16	0.01	0.10	**2.03**

The acronym ND (not determined) was used when the concentration at the numerator or denominator (or both) was below the limit of quantification). In bolt, the values are higher than 1000 for BAF and higher than 1 for BSAF. *SLR* San Leonardo River, *GB* Gorgo Basso, *CR* Cuccumella Reservoir, CR.

In general, BAF was classified as less bioaccumulative (BAF < 1000), bioaccumulative (1000 < BAF < 5000) and highly bioaccumulative (BAF > 5000) (Noman et al. 2022). In the majority of cases, BAF values are below the bioaccumulation criteria, which is likely due to the levels of environmental contamination that are relatively low to result in the high absorption or adsorption of these trace elements in *P. clarkii*. However, there are some notable exceptions. Following these criteria were bioaccumulative: Fe in muscle from GB, Ni in muscle from SLR and Ba in exoskeleton from SLR and GB; instead, BAF showed high accumulative potential for Fe in muscle and exoskeleton from SLR and muscle samples from GB; in addition, BAF was higher than 5000 for Mn in all samples and sites except for muscle from GB and for barium in exoskeleton in all the sites.

The elevated manganese levels in the exoskeleton may be indicative of a high degree of bioaccumulation from water, potentially due to the incorporation of manganese into the calcium carbonate structure (Abbas et al. 2023).

The BSAF was used to evaluate metal transfer from sediment to *P. clarkii*. As observed for other aquatic organisms, BSAF values of < 1, 1–2 and > 2 for *P. clarkii* could indicate deconcentration, microconcentration and macroconcentration status, respectively (Abbas et al. 2023; Ben-Haddad et al. 2023).

In the present work, only five elements had BSAF levels higher than the unit. These values concerned Mn in the exoskeleton from SLR; Cu in all muscle samples and in the exoskeleton from SLR; Zn for GB and SLR muscles; Ba for all exoskeleton samples; and finally, mercury, except for the 1.01 value observed for the LC exoskeleton, it presented the highest BSAF values in the *P. clarki* muscles from the three sites (8.25–15.18). In accordance with the results obtained in the majority of cases, the BSAF values are lower than the unit, indicating that the organism is de-concentrating the metal and releasing it into the sediment. In contrast, the muscular tissues of *P. clarkii* are macroconcentrators of mercury from sediments and, in some cases, copper and zinc. Conversely, the exoskeleton has been observed to macro-concentrate copper (in one case out of three) and barium (in two cases out of three). The BSAF values of Cu and Zn were similar to those observed by Goretti et al. (2016) and Mistri et al. (2020) in Italy. In the latter research, except for arsenic for one site, they were the only two elements to have BSAF values greater than the unit (Mistri et al. 2020). In a similar way to that observed with Abbas et al. (2023), the BSAF values were found to be greater than the BAF values for copper. This suggests that, despite the high concentrations of copper naturally present in *P. clarkii* tissues, the bioaccumulation of Cu in tissues increases in response to significantly higher environmental concentrations which were significantly higher in sediments than in water. Except for the essential and constitutive elements found in *Procambarus clarkii*’s matrices, non-essential elements such as mercury draw attention. The Louisiana red swamp crayfish is known to have close contact with sediment, being a benthic animal and a skilled digger (Souty-Grosset et al. 2016), and it is reasonable to assume that the contribution of mercury from diet or sediment could result in contamination as demonstrated for other trace elements by Rowe et al. (2001). Further investigations will therefore be needed to determine what the sources of such pollution might be, such as the analysis of its potential prey.

Environmental factors such as the specific composition of the sediments and the metabolic characteristics of *P. clarkii* have resulted in the differential distribution of trace elements between muscle and exoskeleton. In this context, non-essential metals do not participate in metabolic processes and their presence in tissues is not regulated, increasing concerns about food safety. In any case, considering the other studies in the literature and the current legislation for heavy metals levels in crustacean meat, the concentration levels recorded in both meat and exoskeleton are of less concern. Based on the obtained results, the *Procambarus clarkii* individuals removed in the frame of monitoring, managing or eradication activities requested by European Community and Italian laws (Tricarico and Zanetti 2023; accessed on 15 January 2024) could be used as a valuable resource to produce feeds, animal meal, biotechnological products, etc. Instead of constituting a waste to be disposed of at extra cost, the obtained carcasses could thus help covering the costs of the management of this invasive species, and this might be applicable also to other invasive decapod species in Sicilian inland waters such as *Callinectes sapidus* and *Cherax destructor*—see Vecchioni et al. 2022b, c), making the implementation of the necessary actions more economically sustainable to local management authorities.

## Conclusions

This study confirmed the potential of *P. clarkii* as an indicator of environmental contamination by validating mild TE pollution levels at the three Sicilian sites. The PERMANOVA, HCA and PCA analyses demonstrated significant discrepancies in contamination profiles between the matrices under examination and between the various sites. In comparison to the results obtained in other literature studies, the levels of contamination recorded in the frame of present research are similar or even lower, showing the occurrence of just a slight environmental contamination in the study sites. The results of the bioaccumulation potentials show that the exoskeleton bioaccumulates more iron, manganese and barium than the muscle, which has a higher concentration of mercury and copper than the exoskeleton. In most cases, BAF and BSAF are below the bioaccumulation criteria. The exceptions are high BAFs for iron and nickel in SLR, manganese in SLR and CR and barium in CR, while high bioaccumulation from sediments was found for mercury and copper in SLR. However, for both mercury and other toxic elements, according to the thresholds reported in the European regulation 2023/915, analyses of Louisiana red swamp crayfish tissues do not give rise to food safety concerns.

However, in this context, it is essential to continue to carry out biomonitoring activities for these and other pollutants throughout the occurrence areas of the species, to confirm these results in the long term, and to promote policies and regulations of environmental sustainability, such as the eradication of *P. clarkii*; this would be of particular importance for the most threatened ecosystems, to improve the quality of life of their components.

## Supplementary Information

Below is the link to the electronic supplementary material.

Supplementary file1 (PDF 4.85 MB)

## Data Availability

All data generated or analysed during this study are included in this published article and its supporting information files.
