# Supplementary material for: Investigating heavy metals and other elements in Procambarus clarkii and environmental matrices from three wetlands of Sicily (Italy)
Source: Environ Sci Pollut Res Int. 2025 Jan 31;32(8):4754–70. doi: 10.1007/s11356-025-35954-y (PMC11850539; doi:10.1007/s11356-025-35954-y)
Supplement: Supplementary file 1 — Supplementary file1 (PDF 4.85 MB) [file 11356_2025_35954_MOESM1_ESM.pdf]

Supporting Information

Investigating heavy metals and other elements in *Procambarus clarkii* and environmental matrices from three wetlands of Sicily (Italy)

Dario Savoca[1,2,*], Federico Marrone[1] , Francesco Paolo Faraone[1], Vittoria Giudice[3], Salvatore Messina[3], Gaetano D'Oca[3], Vincenzo Arizza[1,2], Antonella Maccotta[1,2], Luca Vecchioni[1]

[1] Department of Biological, Chemical and Pharmaceutical Sciences and Technologies (STEBICEF), University of Palermo, 90123 Palermo, Italy

[2] NBFC, National Biodiversity Future Center, 90133 Palermo, Italy

[3] ARPA Sicilia, Agenzia Regionale Protezione Ambiente, UOC L2, via Nairobi, 90129 Palermo, Italy

* Corresponding author: Dario Savoca (email: dario.savoca@unipa.it)

Total SI- Tables: 4

Details of sampling areas

The three investigated sites are permanent, freshwater ecosystems. Gorgo Basso (coordinates: 37.609876 N, 12.654905 E) is a permanent pond of natural origin, located within the Nature Reserve “Lago Preola e Gorghi Tondi”. Its watershed is located in a landscape dominated by olive groves, vineyards, and non-irrigated arable land, along with sparsely vegetated areas and natural vegetation. It is located within the N2K Site of Community Importance (ITA010005), in the N2K Special Protection Area (ITA010031), and included among the Important Bird and Biodiversity Areas in the list of Ramsar Zones since 2011. San Leonardo River (coordinates: 37.905663 N, 13.609423 E) is a permanent river, whose hydrographical basin is dominated by non-irrigated arable land and olive groves, along with a significant presence of sclerophyllous vegetation and broad-leaved forests. San Leonardo River has been dammed few kilometers downstream of the sampled site to build the large, man-made “Rosamarina” reservoir. The Cuccumella Reservoir (coordinates: 37.358650 N, 14.927290 E) is an artificial pond built to serve the adjacent organic rice fields. Close to the rice fields, the landscape is dominated by fruit trees plantations, non-irrigated arable land, and sparsely vegetated areas.

Table S1 Sampling information and physical chemical parameters recorded using probes, test strips and colorimetric methods.

**Site**	**Gorgo Basso**	**San Leonardo River**	**Cuccumella Reservoir**
**Date**	24/07/2023	17/07/2023	28/07/2023
**Number of specimens analysed**	118	65	183
**Number of water sample analysed**	3	3	3
**Number of sediment sample analysed**	3	3	3
**Water temperature**	28.6 °C	22 °C	28.7 °C
**Total dissolved solids**	-	-	684 mg L^{-1}
**Conductivity**	4.66 ms	-	-
**Nitrite (NO_2-)**	0	0	0
**Nitrate (NO_3-)**	0	0	0
**General Hardness (GH)**	16 dH°	8-16 dH°	16 dH°
**Carbonate hardness (KH)**	15-20 dH°	20° dH°	15-20 dH°
**pH**	8	8	8-8.4
**Cl_2**	0 mg L^{-1}	0 mg L^{-1}	0 mg L^{-1}

Table S2 Information about pools and *Procambarus clarkii* individuals

Date	Site	Sex	Maturity	Glair glands	weight (g)	total length (cm)	cephalothorax length (cm)	left claw length (cm)	right claw length (cm)	cephalothorax width	abdomen width	right claw width	left claw width	wet weight muscle (g)	Pool number
17/07/2023	SLR	M	yes	no	37.2	11.1	5.7	6.13	4.66	2.74	1.88	1.9	1.49	5	1
17/07/2023	SLR	F	no	no	24.1	9.53	4.99	3.42	3.4	2.26	1.8	1.16	1.15	3.2	1
17/07/2023	SLR	M	yes	no	34.4	10.1	5.16	4.63	4.47	2.4	1.86	1.59	1.54	3.5	1
17/07/2023	SLR	M	yes	no	38.1	10.53	5.25	5.21	5.5	2.5	1.7	1.72	1.62	3.8	1
17/07/2023	SLR	F	no	no	24.5	9.9	5	4.2	3.15	2.32	1.78	1.1	1.11	4	1
17/07/2023	SLR	M	yes	no	38.6	9.94	5.13	5.24	4.23	2.43	1.72	1.24	1.92	3.3	1
17/07/2023	SLR	M	yes	no	42.8	10.24	5.54	5.98	3.65	2.55	2	1.05	2	4.8	1
17/07/2023	SLR	M	yes	no	27.9	9.1	4.7	4.51	4.46	2.21	1.76	1.34	1.4	3	1
17/07/2023	SLR	M	yes	no	19.2	8.48	4.49	3.44	3.53	1.99	1.48	1.08	1.09	1.9	1
17/07/2023	SLR	M	yes	no	31.3	9.57	4.93	4.3	4.42	2.38	1.6	1.46	1.22	3.4	1
17/07/2023	SLR	M	yes	no	37.4	10.4	5.33	4.92	5	2.38	1.91	1.5	1.4	3.3	1
17/07/2023	SLR	F	no	no	23.1	9.18	4.8	3.12	3.16	2.13	1.8	1.03	1.02	2.5	1
17/07/2023	SLR	M	yes	no	38	10.46	5.45	4.28	4.32	2.61	1.89	1.21	1.25	3.6	1
17/07/2023	SLR	M	yes	no	61.7	12.04	6.16	4.45	7.44	2.95	1.98	2.21	1.07	4.3	2
17/07/2023	SLR	F	yes	yes	29.1	9.81	5.06	3.25	3.25	2.39	1.86	1.11	1.13	3.3	2
17/07/2023	SLR	M	yes	no	19.8	8.68	4.34	3.64	3.69	1.93	1.45	1.19	1.16	2.8	2
17/07/2023	SLR	M	yes	no	27.8	9.91	5.12	4.36	4.33	2.35	1.79	1.14	1.17	3.4	2
17/07/2023	SLR	M	yes	no	17	8.62	4.33	3.09	3.04	1.96	1.44	0.8	0.87	2.2	2
17/07/2023	SLR	F	no	no	29.6	10.12	5.12	3.17	3.25	2.31	1.9	1.19	1.14	4.1	2
17/07/2023	SLR	F	yes	yes	28.4	9.95	5.29	1.67	3.49	2.31	2	1.32	0.47	4.3	2

17/07/2023	SLR	M	si	no	12.5	7.44	3.82	2.87	2.87	1.63	1.41	0.83	0.86	1.9	2
17/07/2023	SLR	M	si	no	40.6	11.48	6.17	missing	missing	2.64	1.94	missing	missing	5.6	2
17/07/2023	SLR	F	no	no	27.2	9.98	5.04	3.16	3.14	2.5	1.88	1.08	1.05	3.6	2
17/07/2023	SLR	M	yes	no	49.1	11.61	5.66	5.84	4.94	2.52	1.99	1.43	2.07	4.7	2
17/07/2023	SLR	F	yes	yes	29.7	10.33	5.02	3.2	3.24	2.37	1.76	1.18	1.2	4.3	2
17/07/2023	SLR	M	yes	no	20.7	9.28	4.6	3.74	3.73	1.93	1.47	1.12	1.13	2.8	2
17/07/2023	SLR	F	yes	no	38.46	10.73	5.66	3.54	3.98	2.53	1.86	1.41	1.17	4.9	3
17/07/2023	SLR	F	yes	no	39.2	11.32	5.73	4.05	4.16	2.58	2.01	1.47	1.43	4.6	3
17/07/2023	SLR	F	no	no	22.76	9.17	4.73	2.86	2.84	2.13	1.66	1.06	1.05	3.6	3
17/07/2023	SLR	M	no	no	29.7	10.51	5.22	4.1	3.89	2.46	1.82	0.93	1.15	4.7	3
17/07/2023	SLR	M	yes	no	15.9	8.38	4.14	3.24	3.07	1.86	1.49	0.96	0.97	2.2	3
17/07/2023	SLR	M	yes	no	23.79	9.28	4.57	3.74	3.77	2.07	1.71	1.2	1.19	3.1	3
17/07/2023	SLR	M	yes	no	32.2	11.12	5.32	4.93	missing	2.33	1.82	missing	1.52	4.4	3
17/07/2023	SLR	F	yes	yes	37.35	11.32	5.55	3.36	4	2.51	1.78	1.32	1.1	5.1	3
17/07/2023	SLR	M	yes	no	25.34	9.43	4.77	3.7	3.68	2.09	1.69	1.15	1.16	3.3	3
17/07/2023	SLR	F	no	no	27.65	10.14	5.01	3.16	3.07	2.25	1.92	1.11	1.1	3.8	3
17/07/2023	SLR	M	yes	no	37.04	10.71	5.57	5.2	3.98	2.44	1.77	1.08	1.65	3.7	3
17/07/2023	SLR	M	yes	no	33.41	10.17	5.11	4.57	4.5	2.3	1.69	1.45	1.46	3.5	3
17/07/2023	SLR	F	no	no	18.11	8.57	4.3	2.7	2.9	1.94	1.58	0.98	0.94	3	3
17/07/2023	SLR	M	yes	no	44.13	11.82	5.84	5.56	5.55	2.58	2.01	1.74	1.8	5.3	4
17/07/2023	SLR	F	no	no	23.93	9.87	4.76	3.31	3.5	2.23	1.77	1.16	1.18	3	4
17/07/2023	SLR	M	yes	no	50.89	11	5.82	5.55	5.7	2.66	1.95	1.74	1.93	3.9	4
17/07/2023	SLR	M	yes	no	38.66	10.53	5.26	5.1	5.08	2.33	1.75	1.57	1.6	4.2	4
17/07/2023	SLR	M	yes	no	24.37	9.6	4.76	4.1	3.97	2.05	1.58	1.3	1.28	3	4
17/07/2023	SLR	M	yes	no	20.78	9.66	4.75	missing	3.76	1.95	1.52	1.12	missing	3	4
17/07/2023	SLR	F	no	no	18.45	9.12	4.43	2.42	2.79	1.85	1.62	0.97	0.83	2.5	4
17/07/2023	SLR	M	yes	no	48.93	10.97	5.52	5.45	5.47	2.63	2.03	1.76	1.75	4.7	4

17/07/2023	SLR	F	yes	yes	42.41	11.56	5.78	4.06	3.95	2.56	2.17	1.42	1.43	5.6	4
17/07/2023	SLR	M	yes	no	36.03	10.1	5.32	4.61	4.64	2.31	1.9	1.39	1.36	3.8	4
17/07/2023	SLR	F	no	no	21.08	9.21	4.72	missing	3.02	2.15	1.71	1.05	missing	2.9	4
17/07/2023	SLR	M	yes	no	16.29	8.45	4.12	2.97	3.17	1.71	1.54	0.97	0.85	2.5	4
17/07/2023	SLR	M	yes	no	20.76	8.94	4.52	3.64	3.69	1.94	1.6	1.13	1.17	2.4	4
17/07/2023	SLR	M	yes	no	19.13	8.51	4.34	3.56	3.51	1.84	1.53	1.14	1.13	2.1	5
17/07/2023	SLR	M	yes	no	43.48	11.6	5.52	5.16	5.24	2.47	1.96	1.6	1.5	4.1	5
17/07/2023	SLR	M	yes	no	40.14	10.65	5.43	5.11	5.3	2.5	1.95	1.54	1.41	4.5	5
17/07/2023	SLR	M	yes	no	38.76	10.7	5.46	5.27	5.25	2.56	2	1.66	1.65	3.9	5
17/07/2023	SLR	F	yes	yes	36.89	11.44	5.6	3.93	3.41	2.49	2.03	1.2	1.39	4.9	5
17/07/2023	SLR	M	yes	no	23.13	9.44	4.63	4.06	3.99	2.13	1.56	1.22	1.21	2.4	5
17/07/2023	SLR	M	yes	no	22.74	8.88	4.48	4.16	4.06	1.98	1.58	1.13	1.25	2.9	5
17/07/2023	SLR	M	yes	no	17.51	8.7	4.41	3.44	3.5	1.83	1.55	1.02	0.96	2.5	5
17/07/2023	SLR	M	yes	no	42.57	10.94	5.57	5.17	5.05	2.43	1.99	1.64	1.67	4.7	5
17/07/2023	SLR	M	yes	no	35.09	9.76	4.98	4.81	4.66	2.26	1.83	1.52	1.51	3.4	5
17/07/2023	SLR	F	yes	yes	39.64	10.91	5.59	4.12	4.07	2.45	1.97	1.45	1.49	4.4	5
17/07/2023	SLR	F	no	no	23.99	9.75	4.74	3.15	3.11	2.11	1.65	1.06	1.04	3.2	5
17/07/2023	SLR	F	yes	yes	26.56	10.32	4.98	2.92	2.77	2.13	1.91	1.03	1.02	4.1	5
24/07/2023	GB	F	yes	yes	30.8	10.2	5.1	3.9	3.85	2.3	2.01	1.27	1.31	3.1	1
24/07/2023	GB	F	yes	yes	21.1	8.97	4.62	2.92	3.04	2.02	1.11	1.09	1.07	2.2	1
24/07/2023	GB	F	no	no	22.6	9.43	4.61	2.77	3.28	2.14	1.81	1.18	0.75	3.1	1
24/07/2023	GB	F	yes	yes	27.3	10	5.32	3.77	3.03	2.33	1.97	0.87	1.21	3.3	1
24/07/2023	GB	F	no	no	16.3	8.54	4.31	1.6	2.79	1.96	1.64	0.99	0.46	2.6	1
24/07/2023	GB	F	yes	yes	25.6	9.67	4.9	3.16	3.35	2.23	1.82	1.16	1.1	2.5	1
24/07/2023	GB	F	yes	yes	26.2	10.02	5.02	3.81	2.14	2.22	1.91	0.68	1.34	2.9	1
24/07/2023	GB	M	yes	yes	26.1	9.15	4.61	4.49	4.55	2.13	1.12	1.37	1.41	2.3	1
24/07/2023	GB	F	yes	yes	22.5	9.59	4.74	2.94	2.91	2.21	1.84	2.88	2.98	3	1

24/07/2023	GB	F	no	no	17.8	8.89	4.48	2.69	2.82	1.96	1.63	0.93	0.94	2.5	1
24/07/2023	GB	M	yes	yes	14.4	7.89	3.84	3.12	3.06	1.7	1.5	0.94	0.98	2	1
24/07/2023	GB	F	no	no	22.6	9.09	4.66	3.29	3.39	2.08	1.75	1.09	1.06	2.6	1
24/07/2023	GB	M	yes	yes	19.5	8.63	4.4	3.44	3.5	1.94	1.66	1.01	0.87	2.9	1
24/07/2023	GB	F	no	no	15.6	8.27	4.12	2.54	2.55	1.85	1.65	0.87	0.89	1.8	1
24/07/2023	GB	M	si	si	33.9	10.35	5.36	4.77	4.56	2.32	1.96	1.27	1.28	2.9	1
24/07/2023	GB	F	si	si	19.6	9.01	4.39	2.93	2.87	2.05	1.7	0.82	0.89	2.8	1
24/07/2023	GB	F	si	si	21.3	9.27	4.71	3.05	3.19	2.02	1.76	1.05	0.94	2.9	1
24/07/2023	GB	F	si	si	25.5	9.83	4.74	3.52	3.54	2.1	1.84	1.22	1.19	3.1	1
24/07/2023	GB	F	si	si	30.5	10.12	5.06	3.83	3.75	2.32	1.94	1.39	1.23	4.1	1
24/07/2023	GB	F	si	si	31.6	10.36	5.24	2.68	3.62	2.39	2.97	1.3	0.86	4.2	1
24/07/2023	GB	F	si	si	25.7	9.62	4.75	3.66	3.52	2.25	1.83	1.18	1.15	2.8	2
24/07/2023	GB	M	yes	yes	20.3	8.8	4.36	3.99	3.92	2.03	1.62	1.14	1.16	2.5	2
24/07/2023	GB	M	yes	yes	22.6	8.97	4.42	3.82	3.95	1.97	1.64	1.23	1.25	0.9	2
24/07/2023	GB	F	yes	yes	22.2	9.57	4.8	3.29	3.47	2.14	1.78	0.91	1.11	3.1	2
24/07/2023	GB	M	yes	yes	20	8.27	4.4	3.84	3.87	1.96	1.61	1.18	1.17	2.2	2
24/07/2023	GB	F	yes	yes	24.1	9.23	4.66	3.52	3.26	2.17	1.84	1.15	1.21	2.9	2
24/07/2023	GB	M	yes	yes	19.7	8.39	4.43	3.87	3.91	1.91	1.67	1.16	1.12	2.5	2
24/07/2023	GB	M	yes	yes	23.6	8.97	4.56	4.31	4.2	2.06	1.68	1.4	1.41	2.2	2
24/07/2023	GB	M	yes	yes	21.7	9.24	4.77	no	4.38	2.18	1.72	1.33	no	2.9	2
24/07/2023	GB	M	yes	yes	33.1	9.97	5.04	4.89	4.93	2.39	1.88	1.55	1.52	2.4	2
24/07/2023	GB	F	yes	yes	27.9	9.83	4.9	3.88	3.89	2.21	1.96	1.24	1.18	2.3	2
24/07/2023	GB	M	yes	yes	28.9	9.6	5.05	2.96	4.79	2.18	1.84	1.44	0.75	1.8	2
24/07/2023	GB	F	yes	yes	26.4	9.77	4.92	3.55	3.41	2.27	1.86	1.14	1.18	3.7	2
24/07/2023	GB	M	yes	yes	18.2	8.47	4.25	3.78	3.68	1.89	1.62	1.12	1.1	2.1	2
24/07/2023	GB	F	yes	yes	28.9	10.3	5.22	no	3.65	2.3	1.99	1.14	no	2.8	2
24/07/2023	GB	F	yes	yes	24.8	9.75	5.06	3.52	3.28	2.23	1.84	1.08	1.11	3.4	2

24/07/2023	GB	M	yes	yes	34.3	10.12	5.17	4.46	5.06	2.36	1.88	1.56	1.23	3.1	2
24/07/2023	GB	M	yes	yes	19.7	8.86	4.6	3.32	3.91	1.96	1.67	1.09	0.81	2.2	2
24/07/2023	GB	F	yes	yes	24.6	9.55	4.85	3.52	3.53	2.15	1.85	1.24	1.09	3	2
24/07/2023	GB	F	yes	yes	27.8	9.8	5.09	3.58	3.75	2.31	1.92	1.13	1.18	3.1	2
24/07/2023	GB	F	yes	yes	24.6	9.77	4.93	no	3.69	2.27	1.92	1.15	no	3.5	3
24/07/2023	GB	F	yes	yes	21.1	9.16	4.42	2.44	2.68	2.08	1.82	0.78	0.61	2.6	3
24/07/2023	GB	F	yes	yes	28.1	10.22	5.23	3.86	3.69	2.25	1.83	1.11	1.33	3.2	3
24/07/2023	GB	M	yes	yes	25.8	9.56	4.86	4.63	3.31	2.2	1.68	0.92	1.19	1.4	3
24/07/2023	GB	F	yes	yes	16.5	8.4	4.33	2.75	2.79	1.87	1.66	0.94	0.85	2.2	3
24/07/2023	GB	F	yes	yes	24.2	9.39	4.72	3.53	3.41	2.23	1.86	1.15	1.12	3.2	3
24/07/2023	GB	M	yes	yes	23.4	9.03	4.67	3.94	3.83	2.05	1.7	1.2	1.3	1	3
24/07/2023	GB	M	yes	yes	22.8	9.06	4.66	4.24	4.33	2.02	1.7	1.32	1.22	2.7	3
24/07/2023	GB	F	yes	yes	20.6	9.29	4.61	3.22	2.99	2.02	1.8	0.9	0.91	3.2	3
24/07/2023	GB	M	yes	yes	20.4	9.26	4.71	4.33	no	2.03	1.7	no	1.3	2.4	3
24/07/2023	GB	M	yes	yes	26.3	9.58	5.13	4.77	no	2.3	1.83	no	1.45	3.1	3
24/07/2023	GB	M	yes	yes	19.8	9.2	4.21	no	3.71	2.13	1.71	1.05	no	2.8	3
24/07/2023	GB	F	yes	yes	29.4	9.92	5.09	3.56	3.48	2.28	1.94	1.25	1.21	2.9	3
24/07/2023	GB	M	yes	yes	21	8.93	4.48	3.72	3.93	2.03	1.57	1.14	1.11	2.1	3
24/07/2023	GB	M	yes	yes	16.3	8.4	4.19	3.4	3.4	1.8	1.5	1	1	2	3
24/07/2023	GB	M	yes	yes	17.2	8	4.1	3.7	3.6	1.8	1.5	1	1.1	2.2	3
24/07/2023	GB	M	yes	yes	16.2	8.6	4.3	no	3.7	2	1.4	1.1	no	2.5	3
24/07/2023	GB	F	yes	yes	7.7	6.7	3.5	1.8	1.6	1.5	1.4	0.4	0.4	1.6	3
24/07/2023	GB	F	yes	yes	15.9	8.7	4.2	2.8	2.7	1.9	1.5	0.8	0.8	2.9	3
24/07/2023	GB	F	yes	yes	21.6	9	4.3	2.8	3.1	2	1.6	1	0.9	3	3
24/07/2023	GB	M	yes	yes	21.5	8.9	4.5	4.2	no	2.1	1.7	no	1.3	2.6	3
24/07/2023	GB	F	yes	yes	9.7	7.3	3.7	2.2	2	1.6	1.3	0.6	0.6	1.7	3
24/07/2023	GB	F	yes	yes	16.7	8.5	4.2	3.2	2.6	1.9	1.7	0.8	0.6	2.8	3

24/07/2023	GB	F	yes	yes	27.7	10	5.1	3.6	2.6	2.3	1.8	0.8	1.4	3.5	4
24/07/2023	GB	M	yes	yes	20.5	8.6	4.4	3.6	3.6	2.1	1.4	1.2	1	1.9	4
24/07/2023	GB	M	yes	yes	26.1	9.2	4.9	2.4	4.7	2.2	1.7	1.5	0.6	2.1	4
24/07/2023	GB	M	yes	yes	19.1	8.5	4.3	3.8	3.9	2	1.5	1.1	1.1	2.6	4
24/07/2023	GB	M	yes	yes	20.07	8.9	4.6	4	2.2	2	1.7	0.6	1.2	2.8	4
24/07/2023	GB	F	yes	yes	20.3	9	4.6	2.8	2.6	2.1	1.6	0.8	0.8	3	4
24/07/2023	GB	M	yes	yes	17.5	8.5	4.4	2.5	3.6	2	1.6	1.1	0.7	2.6	4
24/07/2023	GB	M	yes	yes	21.3	8.7	4.4	3.9	3.9	2	1.5	1.1	1.2	1.8	4
24/07/2023	GB	F	yes	yes	18.8	8.6	4.2	3.1	3	2	1.5	1	0.9	2.9	4
24/07/2023	GB	M	yes	yes	18.2	8.7	4.3	3.9	2.5	2	1.5	0.7	1.2	2.3	4
24/07/2023	GB	M	yes	yes	14.1	7.8	4	3.2	3	1.8	1.5	0.9	0.9	2	4
24/07/2023	GB	F	yes	yes	15	8.1	4.1	2.9	2.7	1.8	1.5	0.9	0.9	3.2	4
24/07/2023	GB	F	yes	yes	12.9	7.4	4	2.6	2.4	1.7	1.5	0.8	0.8	2.3	4
24/07/2023	GB	M	yes	yes	19.3	8.9	4.5	no	3.5	2.1	1.5	0.9	no	1.5	4
24/07/2023	GB	M	yes	yes	17.8	8.3	4.2	3.5	3.5	1.8	1.4	1.1	1.1	2	4
24/07/2023	GB	M	yes	yes	15.1	8	4.2	3.4	3.2	1.8	1.4	0.9	1	2.3	4
24/07/2023	GB	M	yes	yes	17.5	8.6	4.3	3.8	2.7	1.9	1.5	0.7	1.2	1.1	4
24/07/2023	GB	M	yes	yes	18.8	8.9	4.7	3.9	no	2	1.7	no	1.2	2.1	4
24/07/2023	GB	M	yes	yes	15.7	8.2	4.1	3.3	3.3	1.9	1.5	1	1	1.7	4
24/07/2023	GB	M	yes	yes	20.2	8.8	4.4	3.9	9.7	2.1	1.3	1.3	1.3	3.5	4
24/07/2023	GB	F	yes	yes	20.6	9	4.2	3	2.9	2.1	1.7	1	0.9	2.9	4
24/07/2023	GB	M	yes	yes	20.6	9.2	4.6	3.8	3.8	2	1.6	1.1	1.1	2.1	4
24/07/2023	GB	F	yes	yes	15.1	8.5	4.2	2.7	2.6	1.9	1.6	0.9	0.8	1.9	4
24/07/2023	GB	M	yes	yes	13.2	7.7	3.7	3.1	2.9	1.7	1.4	0.8	0.8	2.3	4
24/07/2023	GB	M	yes	yes	16.5	8.5	4.2	3.3	3.4	1.9	1.5	1	1	2.4	4
24/07/2023	GB	M	yes	yes	16.5	8.3	4.3	2.9	3.9	1.9	1.5	1.1	0.7	2.6	4
24/07/2023	GB	M	yes	yes	20.3	8.8	4.8	4	3.8	2	1.5	1.1	1.2	3.1	4

24/07/2023	GB	F	yes	yes	20.6	9.3	4.6	3.2	3.1	2	1.6	1.1	1.1	3.3	4
24/07/2023	GB	F	yes	yes	30.9	10	5.3	2.9	3.4	2.3	1.8	1.3	1.3	3.4	4
24/07/2023	GB	F	yes	yes	35.5	10.7	5.7	3.4	3.8	2.6	2	1.3	1.1	3.9	5
24/07/2023	GB	M	yes	yes	21.7	8.9	4.5	4.2	4.1	2.1	1.6	1.2	1.2	2.6	5
24/07/2023	GB	M	yes	yes	17.8	8.5	4.4	3.3	3.1	1.9	1.5	0.9	0.8	2.7	5
24/07/2023	GB	M	yes	yes	17.2	8.5	3.2	3.5	1.9	1.5	1	1	0.8	0.9	5
24/07/2023	GB	M	yes	yes	14.9	8.1	3.5	3.4	3.4	1.8	1.4	0.9	0.9	2.2	5
24/07/2023	GB	M	yes	yes	23.9	9.2	4.8	3.4	5.2	2.2	1.6	1.6	0.9	2.4	5
24/07/2023	GB	F	yes	yes	15	8.1	4.2	2.6	2.5	1.9	1.4	0.8	0.7	2.6	5
24/07/2023	GB	M	yes	yes	12	7.9	4	3.1	2.4	1.7	1.3	0.6	0.9	1.7	5
24/07/2023	GB	M	yes	yes	22.1	8.8	4.6	no	4.3	2.1	1.7	1.4	no	2.6	5
24/07/2023	GB	F	yes	yes	24.1	9.3	4.8	3.2	3.4	2.2	1.7	1.2	1	3.4	5
24/07/2023	GB	F	yes	yes	31.2	10.2	5.4	3.1	3.4	2.5	2	1.1	0.9	4.8	5
24/07/2023	GB	M	yes	yes	22.1	8.7	4.7	3.5	3.6	2.1	1.6	1	1	3.1	5
24/07/2023	GB	M	yes	yes	20.1	9.1	4.4	2.2	2.6	2.2	1.6	1	0.6	2.7	5
24/07/2023	GB	M	yes	yes	22.9	9.3	4.9	no	no	2.3	1.7	no	no	3	5
24/07/2023	GB	M	yes	yes	13.3	8.7	4.4	3.2	3.8	1.9	1.5	1.2	0.8	2.3	5
24/07/2023	GB	M	yes	yes	18.2	8.8	4.7	3.1	3.4	1.9	1.5	1.1	1	2.7	5
24/07/2023	GB	M	yes	yes	15.9	7.9	4.1	3	3.2	1.9	1.4	1	0.7	2.6	5
24/07/2023	GB	M	yes	yes	14.3	8.1	4	2.9	3	1.8	1.5	0.8	0.8	2.1	5
24/07/2023	GB	F	yes	yes	26.4	9.8	5	3.3	3.3	2.4	1.8	1.1	1	3.5	5
24/07/2023	GB	M	yes	yes	20.7	8.5	4.4	4	3.7	2	1.5	1.2	1.2	1.9	5
24/07/2023	GB	M	yes	yes	17.7	8.4	4	3.5	3.3	1.8	1.4	1	1	2.7	5
24/07/2023	GB	M	yes	yes	14.8	8.3	4.2	2.8	3.6	1.9	1.4	1.1	0.7	1.3	5
24/07/2023	GB	M	yes	yes	17.8	8.3	4.1	3.5	3.6	2	1.6	1	1	2.5	5
24/07/2023	GB	F	yes	yes	19.4	8.8	4.5	3.1	3	1.9	1.6	1	1.1	3	5
24/07/2023	GB	M	yes	yes	24	9.5	4.8	3.3	4.5	3.1	1.7	1.3	0.8	3.4	5

24/07/2023	GB	F	yes	yes	25.7	9.9	5	3.1	3.1	2.3	1.8	1	1	3.8	5
28/07/2023	CR	F	yes	yes	29.29	9.83	5.09	3.83	3.37	2.25	1.94	1.1	1.19	4.3	1
28/07/2023	CR	M	yes	no	17.29	8.18	4.18	3.43	3.39	1.81	1.55	1.06	1.02	2	1
28/07/2023	CR	M	yes	no	18.86	8.47	4.23	3.65	3.58	1.89	1.57	1.11	1.12	2.3	1
28/07/2023	CR	M	yes	no	20.45	8.6	4.43	3.87	3.93	1.85	1.59	1.22	1.14	2.4	1
28/07/2023	CR	F	no	no	12.85	7.8	3.9	2.27	2.43	1.72	1.44	0.83	0.81	1.8	1
28/07/2023	CR	M	yes	yes	15.56	7.75	3.95	3.33	3.36	1.76	1.51	1.03	0.91	2.1	1
28/07/2023	CR	M	yes	no	15.85	7.89	4.09	2.74	3.77	1.84	1.47	1.24	0.68	1.5	1
28/07/2023	CR	F	no	no	13.93	8.12	4.17	2.38	2.27	1.76	1.45	0.72	0.8	1.5	1
28/07/2023	CR	M	yes	no	13.1	7.64	4.13	2.72	2.89	1.78	1.45	0.78	0.7	1.7	1
28/07/2023	CR	M	yes	no	12.25	7.66	3.74	3.32	2.4	1.64	1.41	0.6	0.9	1.7	1
28/07/2023	CR	F	yes	yes	25.13	9.95	4.93	2.79	3.47	2.06	1.87	1.13	0.88	3.6	1
28/07/2023	CR	M	yes	yes	13.79	7.71	3.96	3.19	3.19	1.76	1.32	1.04	1.05	1.5	1
28/07/2023	CR	F	no	no	12.56	7.59	3.89	2.39	2.38	1.76	1.47	0.9	0.87	2.1	1
28/07/2023	CR	F	no	no	12.56	7.72	3.92	2.39	2.28	1.73	1.52	0.89	0.88	1.7	1
28/07/2023	CR	M	yes	no	12.64	7.78	3.93	no	3.12	1.66	1.41	1.04	no	1.6	1
28/07/2023	CR	F	yes	yes	24.58	9.68	4.85	3.3	3.33	2.18	1.79	1.12	1.22	3	1
28/07/2023	CR	M	yes	no	15.73	8.19	4.11	3.45	3.24	1.83	1.55	0.9	1.11	1.5	1
28/07/2023	CR	F	yes	yes	25.27	9.92	4.91	3.63	2.7	2.11	1.82	0.9	1.32	3.6	1
28/07/2023	CR	F	yes	yes	25.64	9.8	4.83	3.13	3.12	2.13	1.93	1.06	1.04	3.5	1
28/07/2023	CR	F	yes	yes	21.21	9.33	4.68	3.02	3.03	2.03	1.66	1	1.01	2.7	1
28/07/2023	CR	M	yes	no	21.76	8.78	4.52	4	4	1.97	1.6	1.29	1.21	0.9	1
28/07/2023	CR	F	no	no	26.63	9.92	5.03	2.89	3.22	2.34	1.89	0.89	0.82	2.6	1
28/07/2023	CR	M	yes	no	15.93	8.07	4.06	3.4	3.25	1.77	1.46	1.05	1.04	2.6	1
28/07/2023	CR	M	yes	no	11.48	7.72	3.74	2.92	2.81	1.65	1.28	0.71	0.82	1.5	1
28/07/2023	CR	M	yes	no	13.62	7.78	3.94	3.04	3.02	1.73	1.43	0.95	0.92	1.8	1
28/07/2023	CR	M	yes	no	33.84	10.63	5.49	3.49	4.23	2.31	1.95	1.33	1.05	4.8	1

28/07/2023	CR	F	yes	yes	25	9.82	4.95	3.33	2.87	2.21	1.72	0.91	1.22	3.9	1
28/07/2023	CR	M	yes	no	12.19	7.78	3.86	2.94	2.92	1.65	1.32	0.94	0.93	1.5	1
28/07/2023	CR	F	no	no	11.95	7.87	3.77	2.26	2.34	1.63	1.47	0.81	0.86	1.8	1
28/07/2023	CR	F	no	no	9.28	7.63	3.75	1.57	1.58	1.54	1.37	0.4	0.4	1.6	1
28/07/2023	CR	F	no	no	12.97	7.98	4.02	2.55	1.87	1.77	1.49	0.64	0.89	1.7	1
28/07/2023	CR	F	no	no	16.29	8.83	4.21	2.84	2.88	1.84	1.58	1.09	0.99	2	1
28/07/2023	CR	F	no	no	14.45	8.34	4.1	2.42	2.63	1.8	1.56	0.94	0.86	2.1	1
28/07/2023	CR	M	yes	no	16.53	7.89	3.97	3.3	3.36	1.79	1.38	1.15	1.14	1.8	1
28/07/2023	CR	M	yes	no	14.27	8.15	3.95	3.16	3.17	1.76	1.44	1.01	0.92	1.9	1
28/07/2023	CR	F	no	no	24.21	9.61	4.71	3.17	3.1	1.95	1.68	1.16	1.12	3.6	1
28/07/2023	CR	F	no	no	10	7.82	3.82	1.95	1.88	1.57	1.36	0.57	0.52	2.3	1
28/07/2023	CR	F	no	no	9.25	7.04	3.41	2.02	2.01	1.48	1.36	0.64	0.62	2.3	1
28/07/2023	CR	M	yes	no	27	9.21	4.75	4.41	4.28	2	1.7	1.46	1.46	2.3	1
28/07/2023	CR	M	yes	no	15.49	8.13	4.05	3.43	3.35	1.72	1.47	1.05	1.07	1.6	1
28/07/2023	CR	M	yes	no	18.47	8.3	4.22	3.63	3.47	1.82	1.54	1.1	1.1	2.8	1
28/07/2023	CR	M	yes	no	11.96	7.45	3.7	2.88	2.87	1.66	1.34	0.98	0.88	1.1	1
28/07/2023	CR	M	yes	no	15.82	8.2	4.18	3.36	3.29	1.71	1.37	1.02	1	1.4	1
28/07/2023	CR	F	no	no	11.52	8.08	3.96	2.42	2.47	1.75	1.82	0.83	0.71	1.9	1
28/07/2023	CR	F	no	no	15.56	8.43	4.28	2.27	2.53	1.83	1.47	0.85	0.92	3.1	1
28/07/2023	CR	F	yes	yes	25.82	10.06	5.01	2.23	3.67	2.07	1.78	1.25	0.63	3.7	1
28/07/2023	CR	M	yes	no	22.45	9.6	4.68	3.25	4.41	2.12	1.59	1.29	0.86	3.3	1
28/07/2023	CR	M	yes	no	30.4	10.16	5.21	5.02	2.62	2.23	1.92	0.61	1.59	4.4	1
28/07/2023	CR	M	yes	no	30	9.76	4.95	4.7	4.07	2.17	1.74	1.01	1.52	3.2	1
28/07/2023	CR	M	yes	no	16.67	8.3	4.17	3.51	3.55	1.8	1.51	1.03	1.03	2.8	1
28/07/2023	CR	F	no	no	11.37	7.73	3.83	2.28	no	1.67	1.39	no	0.73	1.8	1
28/07/2023	CR	M	yes	no	30.43	9.63	5.02	4.87	4.9	2.04	1.77	1.43	1.42	3.6	1
28/07/2023	CR	M	yes	no	16.03	8.6	4.38	3.17	3.39	1.74	1.54	1.05	0.98	2.2	2

28/07/2023	CR	F	no	no	13.32	8	3.96	2.66	2.63	1.8	1.39	0.97	0.96	1.6	2
28/07/2023	CR	M	yes	no	15.49	8.23	4.2	3.24	3.25	1.73	1.5	1.02	1	1.5	2
28/07/2023	CR	F	no	no	10.22	7.6	3.84	2.29	2.26	1.61	1.44	0.76	0.71	1.5	2
28/07/2023	CR	F	no	no	15.15	8.6	4.33	2.64	2.64	1.81	1.57	1.94	1.97	2.5	2
28/07/2023	CR	F	no	no	11.28	8	3.82	2.15	2.33	1.62	1.46	0.72	0.64	1.8	2
28/07/2023	CR	F	no	no	15.4	8.4	4.29	2.6	2.64	1.85	1.5	1.01	1.83	3.1	2
28/07/2023	CR	M	yes	no	26.1	10.53	5.45	no	no	2.29	1.84	no	no	2.8	2
28/07/2023	CR	F	no	no	12.76	8.2	4.04	2.52	1.52	1.7	1.5	0.46	0.74	1	2
28/07/2023	CR	F	yes	yes	28.47	10.07	5.04	3.6	3.61	2.16	1.82	1.24	1.25	3.4	2
28/07/2023	CR	M	yes	no	17.44	8.58	4.24	3.36	3.47	1.7	1.55	1.08	1.02	1.6	2
28/07/2023	CR	M	yes	no	16.67	8.25	4.18	3.49	3.73	1.83	1.45	1.17	0.94	1.7	2
28/07/2023	CR	F	no	no	9.5	7.41	3.64	1.92	2.31	1.47	1.34	0.68	0.54	1.6	2
28/07/2023	CR	M	no	no	21.69	8.55	4.61	4.24	3.4	2	1.57	0.87	1.32	2.1	2
28/07/2023	CR	M	yes	no	11.56	7.37	3.61	2.71	2.77	1.55	1.3	0.92	0.89	1.6	2
28/07/2023	CR	M	yes	no	31.23	9.51	4.97	5.05	3.25	2.27	1.83	0.81	1.49	3.8	2
28/07/2023	CR	F	yes	yes	25.3	9.3	4.8	3.22	3.22	2.12	1.79	1.15	1.16	4	2
28/07/2023	CR	F	no	no	19.87	9.14	4.57	2.84	2.45	2.02	1.65	0.76	1.05	2.4	2
28/07/2023	CR	F	no	no	15.4	8.14	4.1	2.68	2.67	1.88	1.53	0.95	0.8	1.9	2
28/07/2023	CR	M	yes	no	28.26	9.44	4.88	4.52	4.11	2.11	1.71	1.15	1.51	3.4	2
28/07/2023	CR	M	yes	no	13.92	8.06	4.02	2.9	2.84	1.68	1.44	0.87	0.87	2.4	2
28/07/2023	CR	F	no	no	14.5	8.36	4.22	2.9	2.81	1.89	1.43	0.95	1.01	1.5	2
28/07/2023	CR	M	yes	no	15.3	8.67	4.33	no	3.51	1.92	1.52	1.26	no	1.5	2
28/07/2023	CR	F	no	no	13.26	8.2	4.13	2.57	2.28	1.8	1.5	0.6	0.68	1.3	2
28/07/2023	CR	F	no	no	14.7	8.64	4.25	2.54	no	1.86	1.43	no	0.93	2.6	2
28/07/2023	CR	M	yes	no	13.63	8.63	4.37	2.67	no	2	1.4	no	0.68	1.8	2
28/07/2023	CR	M	yes	no	13.65	7.3	3.84	3.11	3.21	1.63	1.44	1.03	0.98	2.1	2
28/07/2023	CR	M	yes	no	19.42	8.72	4.42	3.46	3.45	1.86	1.61	0.96	1.06	2.6	2

28/07/2023	CR	M	yes	no	11.12	7.42	3.58	2.64	2.68	1.6	1.28	0.75	0.67	1.5	2
28/07/2023	CR	M	yes	no	19.86	8.53	4.33	3.7	3.72	1.96	1.54	1.16	1.11	2.9	2
28/07/2023	CR	F	no	no	11.29	7.6	3.69	2.22	2.2	1.58	1.35	1.68	0.65	1.8	2
28/07/2023	CR	F	yes	yes	29	10.15	5.08	3.38	3.46	2.28	1.91	1.2	1.19	4.5	2
28/07/2023	CR	F	no	no	19.31	9.03	4.49	2.94	1.98	1.62	1.6	1.2	1.3	3	2
28/07/2023	CR	M	yes	no	10.6	7.03	3.53	2.63	2.64	1.52	1.33	1.67	1.64	1.5	3
28/07/2023	CR	M	yes	no	19.04	8.51	4.39	3.74	3.68	1.86	1.51	1.1	1.14	0.7	3
28/07/2023	CR	F	no	no	11	7.75	3.61	2.04	2.09	1.61	1.41	0.67	0.7	2.2	3
28/07/2023	CR	M	yes	no	20.12	8.69	4.27	3.7	3.69	1.87	1.58	1.1	1.08	3.1	3
28/07/2023	CR	M	yes	no	12.75	7.82	3.99	no	3.34	1.68	1.38	no	0.99	2	3
28/07/2023	CR	M	yes	no	19.86	8.56	4.26	3.68	3.75	1.93	1.58	1.09	1.17	2.6	3
28/07/2023	CR	M	yes	no	21.35	8.69	4.42	3.58	4.14	1.95	1.56	1.34	0.97	1.8	3
28/07/2023	CR	F	no	no	12.52	7.79	3.83	2.28	2.21	1.66	1.33	0.71	0.71	2.5	3
28/07/2023	CR	F	no	no	16.26	8.25	4.14	2.47	2.44	1.79	1.64	0.79	0.87	2.7	3
28/07/2023	CR	F	no	no	14.89	8.14	4.17	2.77	2.8	1.86	1.47	1.01	0.97	1.8	3
28/07/2023	CR	M	yes	no	13.19	7.72	3.82	3.12	3.1	1.72	1.42	0.95	0.93	2.2	3
28/07/2023	CR	M	yes	no	17.27	8.31	4.18	3.55	3.48	1.84	1.58	0.99	0.99	1.4	3
28/07/2023	CR	F	no	no	15.3	8.2	4.18	2.54	2.53	1.83	1.62	0.84	0.81	2.7	3
28/07/2023	CR	F	no	no	23.29	9.12	4.7	3.17	3.19	2.08	1.83	1.02	1.05	3.1	3
28/07/2023	CR	M	yes	no	21.95	8.6	4.4	3.93	3.93	1.94	1.55	1.23	1.25	3.3	3
28/07/2023	CR	F	yes	yes	28.05	9.55	4.75	4.22	4.31	2.12	1.59	1.31	1.36	3.6	3
28/07/2023	CR	F	no	no	12.34	7.54	3.73	2.34	2.35	1.67	1.47	0.8	0.8	1.5	3
28/07/2023	CR	F	no	no	13.64	8.46	4.13	2.11	2.16	1.66	1.52	0.67	0.66	1.8	3
28/07/2023	CR	F	no	no	16.95	8.63	4.31	2.84	1.86	1.83	1.5	1.03	1.01	2	3
28/07/2023	CR	M	yes	no	22.36	8.7	4.6	4.06	4.05	1.99	1.64	1.21	1.19	2.7	3
28/07/2023	CR	M	yes	no	28.93	9.74	4.89	4.96	2.48	2.14	1.77	0.58	1.62	3.4	3
28/07/2023	CR	M	yes	no	17.76	8.26	4.02	3.81	3.84	1.79	1.46	1.23	1.22	1.7	3

28/07/2023	CR	F	no	no	20.47	9	4.46	3.29	3.24	2.06	1.66	1.14	1.13	1.8	3
28/07/2023	CR	M	yes	no	14.17	7.75	3.83	3.05	3.09	1.71	1.43	0.98	0.96	2	3
28/07/2023	CR	M	yes	no	17.53	8.61	4.38	1.83	3.49	1.82	1.43	1.16	0.57	1.5	3
28/07/2023	CR	M	yes	no	12.67	7.91	3.89	3.04	3.03	1.53	1.4	0.87	0.83	2.1	3
28/07/2023	CR	F	no	no	14.44	8.1	4.01	2.44	2.44	1.76	1.6	0.83	0.82	1.8	3
28/07/2023	CR	M	yes	no	10.75	7.44	3.55	2.53	2.52	1.49	1.3	0.82	0.79	1.6	4
28/07/2023	CR	F	no	no	16.46	8.29	4.09	2.68	2.72	1.83	1.59	0.82	0.97	2	4
28/07/2023	CR	M	yes	no	12.12	7.37	3.61	2.27	2.78	1.58	1.34	0.91	0.88	1.6	4
28/07/2023	CR	M	yes	no	11.16	7.42	3.51	2.72	2.84	1.51	1.32	0.89	0.88	1.5	4
28/07/2023	CR	M	yes	no	15.35	8.14	4.07	3.49	3.4	1.79	1.33	1.11	1.11	1.3	4
28/07/2023	CR	M	yes	no	20.28	8.74	4.39	3.91	3.3	1.94	1.59	0.89	1.33	2.1	4
28/07/2023	CR	M	no	no	9.07	6.99	3.43	2.6	2.58	1.37	1.23	0.71	0.75	1.3	4
28/07/2023	CR	M	yes	no	34.86	10.12	5.28	4.82	4.85	2.2	1.85	1.58	1.53	4.4	4
28/07/2023	CR	M	yes	no	27.48	9.63	4.87	3.82	3.68	2.34	1.78	0.93	1.03	2.6	4
28/07/2023	CR	M	yes	no	14.73	7.89	3.83	3.24	3.28	1.75	1.47	0.96	0.9	2.2	4
28/07/2023	CR	M	yes	no	14.95	8.33	4.06	2.99	3.14	1.69	1.45	1.06	0.97	1.8	4
28/07/2023	CR	M	yes	no	20	8.49	4.33	3.79	3.84	1.88	1.49	1.34	1.32	1.8	4
28/07/2023	CR	F	no	no	14.73	8.01	4.11	2.64	2.69	1.84	1.41	1.01	0.98	1.9	4
28/07/2023	CR	M	yes	no	20	8.53	4.31	3.92	4	1.88	1.56	1.31	1.22	2	4
28/07/2023	CR	M	yes	no	13.24	7.74	3.84	3.03	3.07	1.69	1.46	0.93	0.95	2	4
28/07/2023	CR	M	yes	no	9.93	7.13	3.45	2.65	2.6	1.48	1.29	0.79	0.85	1.2	4
28/07/2023	CR	M	yes	no	13.24	7.96	3.85	3.24	3.19	1.59	1.31	0.95	0.95	2.3	4
28/07/2023	CR	M	yes	no	12.81	8.25	4.04	3.48	no	1.74	1.52	no	1.08	1.8	4
28/07/2023	CR	M	yes	no	11.01	7.38	3.64	2.65	2.62	1.47	1.36	0.87	0.81	1.5	4
28/07/2023	CR	F	no	no	13.24	7.97	3.88	2.46	2.47	1.73	1.5	0.83	0.86	1.8	4
28/07/2023	CR	F	no	no	12.57	8.01	3.98	2	2.02	1.78	1.49	0.59	0.59	2.4	4
28/07/2023	CR	M	no	no	12.4	7.75	3.7	2.15	2.87	1.7	1.3	0.91	0.63	1.5	4

28/07/2023	CR	M	yes	no	15.38	8.04	3.98	3.3	3.25	1.73	1.47	1.02	1.01	2.3	4
28/07/2023	CR	M	yes	no	22.45	8.95	4.47	3.83	4.15	1.95	1.61	1.23	1.08	2.9	4
28/07/2023	CR	F	no	no	17.81	8.7	4.27	2.94	2.96	1.87	1.6	0.88	0.95	2.8	4
28/07/2023	CR	M	yes	no	22.41	8.83	4.38	3.92	3.9	1.94	1.63	1.28	1.31	2.4	4
28/07/2023	CR	F	no	no	14	7.86	3.9	2.67	2.66	1.8	1.45	0.91	94	1.8	4
28/07/2023	CR	F	no	no	14.74	8.25	4.02	2.71	2.7	1.77	1.37	0.96	0.94	1.8	4
28/07/2023	CR	F	yes	yes	37.15	10.9	5.54	3.86	4.03	2.48	2.1	1.34	1.37	6.2	4
28/07/2023	CR	M	yes	no	14.53	8.35	4.21	3.45	no	2.34	3.5	no	1.04	2.2	4
28/07/2023	CR	M	yes	no	18.9	8.44	4.23	3.56	3.63	1.91	1.6	1.09	1.11	2.8	4
28/07/2023	CR	M	yes	no	12.51	7.39	3.83	2.97	3.02	1.51	1.37	0.87	0.88	1.7	4
28/07/2023	CR	M	yes	no	14.66	7.72	3.93	3.23	3.17	1.71	1.45	1.05	1.05	1.6	5
28/07/2023	CR	M	yes	no	13.83	8.03	3.97	2.87	2.91	1.69	1.44	0.92	0.91	1.7	5
28/07/2023	CR	F	no	no	19.79	8.82	4.59	2.97	3.01	1.97	1.73	1.11	1.09	2.9	5
28/07/2023	CR	M	yes	no	22.89	8.87	4.58	4.26	3.33	2	1.68	0.89	1.31	2.9	5
28/07/2023	CR	M	yes	no	13.06	7.54	3.74	3.1	3.08	1.6	1.38	1.02	1	1.3	5
28/07/2023	CR	M	yes	no	13.06	7.74	3.84	2.85	2.82	1.68	1.44	0.88	0.84	1.7	5
28/07/2023	CR	M	yes	no	16.85	8.16	4.03	3.28	3.32	1.87	1.44	1.1	1.05	2.2	5
28/07/2023	CR	M	yes	no	15.93	8	4.08	3.31	3.4	1.79	1.5	1.07	1.02	2.1	5
28/07/2023	CR	F	no	no	16.07	8.69	4.27	2.01	2.92	1.86	1.61	1.04	0.58	2	5
28/07/2023	CR	M	yes	no	18.73	8.66	4.39	3.64	3.7	1.88	1.57	1.1	1	2.7	5
28/07/2023	CR	M	yes	no	10.24	7.26	3.53	2.36	2.44	1.44	1.3	0.8	0.74	1.7	5
28/07/2023	CR	M	yes	no	15.22	7.77	3.99	3.13	3.19	1.71	1.51	0.97	1	2.3	5
28/07/2023	CR	M	yes	no	21.3	8.96	4.56	4.34	3.78	1.96	1.57	1.01	1.39	2.2	5
28/07/2023	CR	F	no	no	12.06	7.74	3.89	1.65	2.61	1.65	1.51	0.91	0.44	2.2	5
28/07/2023	CR	M	yes	no	19.31	9.03	4.51	3.21	3.52	1.93	1.64	1.08	0.85	2.3	5
28/07/2023	CR	M	yes	no	14.87	8.14	4.14	2.61	3.35	1.76	1.48	1.14	0.69	2	5
28/07/2023	CR	F	no	no	20.74	8.87	4.33	3.08	3.11	2.07	1.49	1.15	1.15	3.1	5

28/07/2023	CR	M	yes	no	14.72	7.98	3.92	3.06	3	1.77	1.45	0.9	0.94	2.3	5
28/07/2023	CR	M	yes	no	14.44	8.11	4.06	2.76	3.13	1.72	1.47	1.01	0.73	1.9	5
28/07/2023	CR	M	yes	no	15.02	7.79	3.89	3.25	3.24	1.87	1.39	1.05	1.07	1.5	5
28/07/2023	CR	M	yes	no	9.41	7.12	3.42	2.34	2.36	1.49	1.29	0.7	0.73	1.6	5
28/07/2023	CR	M	yes	no	8.98	6.91	3.39	2.24	2.41	1.48	1.24	0.73	0.62	1.6	5
28/07/2023	CR	F	no	no	13.88	8.44	4.16	2.49	1.68	1.81	1.52	0.47	0.81	2.2	5
28/07/2023	CR	M	yes	no	15.13	8	3.98	3.25	3.19	1.75	1.42	0.94	1.04	1.8	5
28/07/2023	CR	M	no	no	11.25	7.87	3.9	2.1	1.77	1.58	1.39	0.46	0.53	1.9	5
28/07/2023	CR	M	yes	no	18.22	8.58	4.42	3.44	3.5	1.92	1.57	1.02	0.91	2.2	5
28/07/2023	CR	M	yes	no	14.1	7.94	3.9	3.16	3.15	1.7	1.4	0.88	0.92	1.9	5
28/07/2023	CR	F	no	no	17	8.42	4.19	2.83	2.58	1.87	1.53	0.77	1.08	2.2	5
28/07/2023	CR	F	no	no	14.1	8.14	4.09	2.14	2.47	1.82	1.54	0.63	0.68	2.1	5
28/07/2023	CR	M	yes	no	14.94	7.99	3.95	3	3.04	1.72	1.45	0.98	1.01	1.9	5
28/07/2023	CR	M	yes	no	16.07	8.03	4.05	3.12	3.17	1.75	1.45	1.05	1.07	1.9	5
28/07/2023	CR	F	no	no	17.22	8.65	4.32	2.95	2.93	1.96	1.47	1.08	1.04	2.2	5
28/07/2023	CR	F	no	no	15.19	8.13	4.04	2.54	2.63	1.83	1.56	0.95	0.98	2.1	5
28/07/2023	CR	M	no	no	14.65	8.73	4.31	2.19	1.34	1.81	1.43	0.38	0.65	2.5	5
28/07/2023	CR	F	no	no	8.81	7.11	3.47	1.84	1.96	1.5	1.37	0.69	0.61	2	5
28/07/2023	CR	M	no	no	10	7.41	3.57	2.48	2.63	1.57	1.32	0.81	0.78	1.3	5
28/07/2023	CR	M	yes	no	22.55	8.58	4.33	4.07	4	1.9	1.61	1.25	1.31	2.8	5
28/07/2023	CR	M	yes	no	14.65	7.63	3.79	3.28	3.32	1.73	1.4	1.11	1.09	1.2	5
28/07/2023	CR	M	yes	no	11.76	7.82	3.9	no	3.04	1.64	1.26	1.06	no	1.4	5

SLR: San Leonardo River; GB: Gorgo Basso; CR: Cuccumella Reservoir

Table S3 Recovery percentage, LOD, average LOQ for each element and matrix

Element	R_{SS}%	R_{CS}%	R_{WS}%	LOD_{SS} and LOD_{CS} (μg g^{-1})	LOQ_{SS} and LOD_{CS} (μg g^{-1})	LOD_{WS} (μg L^{-1})	LOQ_{WS} (μg L^{-1})
Sb	118	118	97	0.10	0.32	0.15	0.5
As	121	85	108	0.10	0.32	0.30	1
B	85	84	90	0.10	0.32	0.30	1
Cd	94	88	97	0.005	0.02	0.0021	0.0071
Co	91	91	99	0.10	0.32	0.30	1
Cr	100	99	95	0.05	0.16	0.15	0.5
Fe	94	85	104	1.34	4.43	4.2	14
Mn	95	85	101	0.29	0.95	0.30	1
Hg	108	85	159	0.001	0.003	0.00003	0.01
Ni	100	117	121	0.10	0.32	0.30	1
Pb	92	92	82	0.10	0.32	0.11	0.37
Cu	93	92	95	0.10	0.32	0.30	1
Se	140	85	119	0.10	0.32	0.30	1
V	117	117	101	0.10	0.32	0.30	1
Zn	106	85	120	0.57	1.91	1.8	6
Ag	N.A.	98	N.A.	0.10	0.32	N.A.	N.A.
Sn	105	105	99	0.10	0.32	0.30	1
Ba	96	96	94	0.10	0.32	0.30	1

R_{SS}: recovery percentage of sediments samples; **R_{CS}** recovery percentage of crayfish samples **R_{WS}** recovery percentage of water samples; **LOD_{SS}**: limit of detection sediments samples; **LOQ_{SS}**: limit of quantification sediment samples; **LOD_{CS}**: limit of detection crayfish samples; **LOQ_{CS}**: limit of quantification crayfish samples; **LOD_{WS}**: limit of detection water samples; **LOD_{QS}**: limit of quantification water samples. **N.A.:** not analysed.

Table S4 Concentration levels (μg g^{-1} d.w. or μg L^{-1} for water).

Sito	Matrix	Pool	Sb	As	B	Cd	Co	Cr	Fe	Mn	Hg	Ni	Pb	Cu	Se	V	Zn	Ag	Sn	Ba
SLR	Muscle	1	<	1.93	1.31	<	<	0.21	16.43	0.75	0.171	0.43	<	18.51	0.42	<	84.72	<	<	0.42
SLR	Muscle	2	<	0.68	0.93	<	<	0.34	58.61	14.11	0.362	0.75	<	57.56	1.31	<	100.25	<	<	0.61
SLR	Muscle	3	<	0.65	1.19	<	<	0.75	179.02	12.19	0.145	7.86	<	75.46	1.51	<	85.00	<	<	0.71
SLR	Muscle	4	<	0.63	1.53	<	<	0.40	153.86	20.26	0.137	0.92	<	63.33	1.30	0.23	80.13	<	<	1.44
SLR	Muscle	5	<	0.71	1.20	<	<	0.44	62.20	10.27	0.298	0.60	<	47.21	1.40	<	94.68	<	<	0.53
GB	Muscle	1	<	2.25	1.59	<	<	0.32	33.84	1.19	0.269	0.46	<	23.17	0.48	<	121.78	<	<	0.60
GB	Muscle	2	<	2.56	1.45	<	<	0.22	31.68	1.38	0.398	<	<	25.09	0.55	<	124.53	<	<	0.72
GB	Muscle	3	<	2.02	1.39	<	<	<	23.15	1.49	0.175	<	<	22.14	0.48	<	75.16	<	<	0.54
GB	Muscle	4	<	2.34	1.47	<	<	0.17	18.57	<	0.280	<	<	29.28	0.49	<	88.62	<	<	0.38
GB	Muscle	5	<	3.00	2.05	<	<	<	38.17	1.34	0.363	0.32	<	35.68	0.56	<	121.16	<	<	0.81
CR	Muscle	1	<	1.77	1.16	<	<	0.49	47.68	11.24	0.320	1.04	<	49.37	1.05	<	113.08	<	<	2.23
CR	Muscle	2	<	1.73	1.39	<	<	0.37	42.85	10.14	0.416	0.66	<	55.80	1.20	<	131.29	<	<	3.23
CR	Muscle	3	<	2.04	1.55	0.02	<	0.38	196.08	7.42	0.278	0.68	<	69.56	1.04	<	107.60	<	<	6.98
CR	Muscle	4	<	1.90	1.65	<	<	1.04	112.14	31.33	0.479	0.36	<	36.23	1.04	<	119.42	<	<	7.90
CR	Muscle	5	<	2.04	1.34	<	<	0.43	58.26	21.88	0.320	0.93	<	56.87	1.10	<	114.32	<	<	8.88
SLR	Exoskeleton	1	<	0.45	3.72	<	1.42	1.01	503.47	528.58	0.020	1.04	<	27.71	0.42	0.93	23.65	<	<	118.52
SLR	Exoskeleton	2	<	0.28	3.94	<	0.99	0.85	394.44	306.42	0.019	1.07	<	27.20	0.36	0.69	13.74	<	<	131.02
SLR	Exoskeleton	3	<	0.62	4.57	<	2.46	1.40	1154.79	801.02	0.015	1.80	0.37	35.47	0.44	1.64	16.38	<	<	182.64
SLR	Exoskeleton	4	<	0.36	5.08	<	1.75	1.21	675.00	571.35	0.009	1.15	<	32.40	0.44	1.29	14.02	<	<	158.36
SLR	Exoskeleton	5	<	0.32	5.00	<	1.38	1.16	627.79	435.77	0.011	0.89	<	24.87	0.39	1.05	11.72	<	<	141.15
GB	Exoskeleton	1	<	0.92	6.65	<	0.59	0.84	169.88	56.00	0.007	0.38	<	14.49	0.33	0.36	16.96	<	<	150.52
GB	Exoskeleton	2	<	0.90	7.28	<	0.67	0.49	176.71	67.51	0.012	0.34	<	12.43	<	0.36	17.49	<	<	129.82
GB	Exoskeleton	3	<	0.67	4.69	<	0.59	0.46	162.75	81.65	0.008	<	<	10.94	0.32	0.37	10.05	<	<	127.94
GB	Exoskeleton	4	<	0.93	5.31	<	0.63	0.64	231.80	56.06	0.012	0.36	<	11.20	0.33	0.55	17.03	<	<	141.41
GB	Exoskeleton	5	<	0.87	6.61	<	0.67	0.64	205.30	81.95	0.008	0.38	<	12.13	0.35	0.44	13.20	<	<	160.79
CR	Exoskeleton	1	<	0.50	4.95	<	0.75	0.68	279.99	158.38	0.008	0.80	<	24.78	0.36	0.77	10.71	<	<	581.09
CR	Exoskeleton	2	<	0.62	5.04	<	0.83	0.63	293.26	139.03	0.009	0.91	<	24.25	0.45	0.79	16.04	<	<	555.36
CR	Exoskeleton	3	<	0.53	4.68	<	0.75	0.67	297.98	173.97	0.009	0.80	<	21.06	0.36	0.78	14.20	<	<	414.64
CR	Exoskeleton	4	<	0.46	5.23	<	1.09	1.05	569.63	268.22	0.011	0.97	<	15.92	0.40	1.56	12.71	<	<	707.86
CR	Exoskeleton	5	<	0.37	5.18	<	0.67	0.67	316.51	120.19	0.007	0.79	<	11.76	0.34	0.84	9.03	<	<	428.31

SLR	Sediments	1	<	7.00	18.94	0.15	9.68	55.07	25394.17	333.88	0.016	19.41	10.08	10.71	1.73	64.13	60.98	<	1.39	127.28
SLR	Sediments	2	<	7.06	11.68	0.11	7.64	30.94	22804.08	373.30	0.018	17.29	6.33	9.45	1.22	39.19	45.42	<	0.72	216.70
SLR	Sediments	3	<	5.48	3.98	0.10	4.59	10.97	15278.72	449.84	0.010	10.12	3.36	4.87	0.65	16.74	26.74	<	<	87.76
GB	Sediments	1	0.51	17.39	13.68	0.12	3.94	18.66	11184.86	170.53	0.032	7.63	10.66	26.46	0.80	35.18	33.92	<	0.51	41.37
GB	Sediments	2	0.61	16.12	18.85	0.15	5.17	17.25	10356.63	215.75	0.038	7.23	15.45	26.07	0.89	33.20	42.54	<	0.65	54.32
GB	Sediments	3	0.54	15.88	17.90	0.13	4.52	16.59	10239.33	191.80	0.038	6.91	11.79	23.85	0.79	32.04	45.08	<	0.58	46.26
CR	Sediments	1	<	9.88	55.88	0.24	17.10	75.32	38416.92	1075.40	0.027	42.57	19.35	31.50	1.99	114.74	120.13	<	2.03	287.13
CR	Sediments	2	<	11.79	62.02	0.28	21.79	97.34	45276.28	982.02	0.028	47.32	24.58	28.56	2.57	144.05	129.86	<	2.72	275.03
CR	Sediments	3	<	7.39	48.77	0.31	19.12	86.86	37965.45	789.32	0.034	44.88	27.89	30.03	2.45	119.50	119.97	<	2.46	232.86
SLR	Water	1	<	<	300.88	<	1.367	<	<	<	<	<	<	<	<	<	<	<	<	32.85
SLR	Water	2	<	<	287.44	<	1.294	<	<	<	<	<	<	<	<	<	<	<	<	33.80
SLR	Water	3	<	<	326.13	0.01	1.509	<	40.617	1.181	<	3.611	<	<	<	<	<	<	<	37.72
GB	Water	1	0.53	10.89	647.38	<	<	<	16.598	<	<	<	<	<	<	1.34	<	<	<	32.13
GB	Water	2	0.52	11.45	630.65	0.01	<	<	18.409	<	<	<	<	<	<	1.32	<	<	<	32.10
GB	Water	3	<	10.58	624.73	<	<	<	24.028	3.811	<	<	<	<	<	<	<	<	<	32.98
CR	Water	1	<	4.50	479.73	0.02	<	<	<	<	<	<	<	<	<	11.16	<	<	<	112.46
CR	Water	2	<	3.56	366.12	<	<	<	<	<	<	<	<	<	<	3.76	<	<	<	18.00
CR	Water	3	<	4.06	413.51	0.01	<	<	<	1.002	<	<	<	<	<	4.23	<	<	<	18.89

SLR: San Leonardo River; GB: Gorgo Basso; CR: Cuccumella Reservoir.
